# Dechloranes and chlorinated paraffins in sediments and biota of two subarctic lakes

**DOI:** 10.3389/ftox.2024.1298231

**Published:** 2024-05-16

**Authors:** Aline Arriola, Insam Al Saify, Nicholas A. Warner, Dorte Herzke, Mikael Harju, Per-Arne Amundsen, Anita Evenset, Claudia Möckel, Ingjerd S. Krogseth

**Affiliations:** ^1^ Akvaplan-niva, Fram Centre, Tromsø, Norway; ^2^ Waternet Institute for the Urban Water Cycle, Department of Technology, Research and Engineering, Amsterdam, Netherlands; ^3^ Thermo Fisher Scientific, Bremen, Germany; ^4^ NILU (Norsk Institutt for Luftforskning), Fram Centre, Tromsø, Norway; ^5^ Department of Arctic and Marine Biology, UiT the Arctic University of Norway, Tromsø, Norway; ^6^ Department of Materials and Environmental Chemistry, Stockholm University, Stockholm, Sweden

**Keywords:** emerging contaminants, chlorinated paraffins, dechloranes, Lakes, sub-arctic

## Abstract

Our understanding of the environmental behavior, bioaccumulation and concentrations of chlorinated paraffins (CPs) and Dechloranes (Dec) in the Arctic environment is still limited, particularly in freshwater ecosystems. In this descriptive study, short chain (SCCPs) and medium chain (MCCPs) CPs, Dechlorane Plus (DP) and analogues, and polychlorinated biphenyls (PCBs) were measured in sediments, benthic organisms, three-spined stickleback (*Gasterosteus aculeatus*), Arctic char (*Salvelinus alpinus*) and brown trout (*Salmo trutta*) in two Sub-Arctic lakes in Northern Norway. Takvannet (TA) is a remote lake, with no known local sources for organic contaminants, while Storvannet (ST) is situated in a populated area. SCCPs and MCCPs were detected in all sediment samples from ST with concentration of 42.26–115.29 ng/g dw and 66.18–136.69 ng/g dw for SCCPs and MCCPs, respectively. Only SCCPs were detected in TA sediments (0.4–5.28 ng/g dw). In biota samples, sticklebacks and benthic organisms showed the highest concentrations of CPs, while concentrations were low or below detection limits in both char and trout. The congener group patterns observed in both lakes showed SCCP profiles dominated by higher chlorinated congener groups while the MCCPs showed consistency in their profiles, with C_14_ being the most prevalent carbon chain length. Anti- and syn-DP isomers were detected in all sediment, benthic and stickleback samples with higher concentrations in ST than in TA. However, they were only present in a few char and trout samples from ST. Dec 601 and 604 were below detection limits in all samples in both lakes. Dec 603 was detected only in ST sediments, sticklebacks and 2 trout samples, while Dec 602 was the only DP analogue found in all samples from both lakes. While there were clear differences in sediment concentrations of DP and Dec 602 between ST and TA, differences between lakes decreased with increasing δ^15^N. This pattern was similar to the PCB behavior, suggesting the lake characteristics in ST are playing an important role in the lack of biomagnification of pollutants in this lake. Our results suggest that ST receives pollutants from local sources in addition to atmospheric transport.

## 1 Introduction

Persistent organic pollutants (POPs) are man-made compounds that have been used extensively in agriculture, industrial and manufacturing processes (Markowitz, 2018; Ontiveros-Cuadras et al., 2019). Due to their physicochemical properties, POPs can undergo long-range atmospheric transport (LRAT), which results in a global distribution and lead to POPs being present in regions far from where they are used and released, such as the Arctic (Hageman et al., 2015; Kallenborn et al., 2015). POPs also have long half-lives and are highly resistant to degradation. This makes them highly persistent in ecosystems and increases the risk for biomagnification in food webs, potentially causing adverse effects in top predators, including humans. While many POPs have been recognized as harmful and have been regulated by the Stockholm Convention since 2004 (Fiedler et al., 2019), new chemicals are continuously introduced in industrial applications, in many cases as replacements for regulated compounds. Around 150,000 chemicals are currently in use by the industry, with many more entering the market every year (AMAP, 2016). Many are organic contaminants with similar properties to legacy POPs (persistent, bioaccumulative, toxic and/or with LRAT potential) but their potential role as environmental contaminants remains poorly understood.

Short and medium chain chlorinated paraffins (SCCPs and MCCPs) and Dechlorane plus (DP) and related compounds (Dec 601, 602, 603, and 604) are examples of contaminants that have 1) recently been regulated (SCCPs, DP), 2) are currently under review for regulation (MCCPs) or, 3) are not regulated (Dec 601–604). Chlorinated paraffins (CPs) are polychlorinated n-alkanes, which can be categorized according to their number of carbons: short chain (SCCPs, C_10-13_), medium chain (MCCPs, C_14-17_) and long chain (LCCPs, C ≥ 18). Differences in carbon chain length and degree of chlorination (30%–70% by weight) give these compounds a wide variety of physico-chemical properties (Glüge et al., 2013). CPs were first introduced on an industrial scale in the 1930s and are now among the most widely produced chemicals in the world (Glüge et al., 2018) with China being the largest producer and consumer (Chen et al., 2021). CPs are stable across a wide range of temperatures and physical conditions, which make them useful in many applications such as in metalworking fluids, in paints and lubricants, as flame retardants, as plasticizers, or as extreme pressure additives (Glüge et al., 2016; Glüge et al., 2018; van Mourik et al., 2016). Despite having been in use for many decades, CPs have only relatively recently been detected in the Arctic environment (Tomy et al., 1999; Tomy et al., 2000; Muir et al., 2000; Reth et al., 2006), mostly due to the development of more sensitive analytical methods and to their inclusion in monitoring programs (Bohlin-Nizzetto and Aas, 2014).

SCCPs have been included in several regulatory bodies in the last years. In Norway, production, sales, import or export of products with more than 0.1% by weight of SCCPs was prohibited in 2002 (ECHA, 2008). SCCPs were included in the Stockholm convention of POPs (Annex A—elimination) in 2017 (UNEP, 2017). While MCCPs have not been regulated, they have been proposed for listing under the Stockholm Convention on persistent organic pollutants (UNEP, 2021). As a result of the regulation of SCCPs, there has been an increase in production and use of MCCPs (Glüge et al., 2018). This is already reflected in the environment, with MCCP mass fractions in biota surpassing those of SCCPs (Vorkamp et al., 2019a).

Dechlorane plus has been in use since the 1960s, first as an insecticide to replace Mirex, and later as a flame retardant additive mainly in wire and electronic appliances (Hoh et al., 2006; Guo et al., 2017; Hansen et al., 2020). It is considered a chemical of high production volume in the US and of low production volume in the EU (Tomy et al., 2007; Wang et al., 2016). DP has two stereoisomers, *syn*-DP and *anti*-DP that are found in an approximate ratio of 1:3 in commercial products (Wang et al., 2016). The isomer ratio has been used to assess the fate and distribution of DP in the environment and it is normally represented as *f*_*anti*_ (*f*_*anti*_ = *anti*DP/(*anti*DP + *syn*DP). In comparison to DP, information on production and use of the related compounds Dec 602, 603, and 604 is very limited (Shen et al., 2011a; Feo et al., 2012).

CPs, DP and Dec 602, Dec 603 have been found in air, water, sediments, and biota in remote areas such as the Arctic and Antarctic (Möller et al., 2010; Möller et al., 2012; Xiao et al., 2012; Vorkamp and Riget, 2014; Vorkamp et al., 2015; Vorkamp et al., 2019a; Vorkamp et al., 2019b; AMAP, 2017; Gao et al., 2018). These chemicals have been measured in top predators, such as whales and seals, indicating biomagnification in the environment (Simond et al., 2017; Vorkamp et al., 2019a). However, the number of studies in freshwater ecosystems is limited to only a few studies in Canada, Sweden and Norway (Schlabach et al., 2017). A review by Vorkamp et al. (2019a) highlights that only 3 studies include MCCPs in fish from Norwegian and Canadian Arctic lakes. Studies including DP in the Arctic/Sub-Arctic focus mainly on marine and terrestrial environments. Given the small number of studies in freshwater ecosystems and the importance of data from the Arctic documenting the LRAT, bioaccumulation, and persistence of contaminants of emerging concern, the 2017 AMAP assessment on contaminants of emerging concern strongly emphasized the need for more information on levels and better understanding of the sources and distribution of these contaminants. Accordingly, this study aims to provide data for CPs, DP and related compounds in freshwater ecosystems in the Norwegian Sub-Arctic. We specifically address the potential biomagnification by sampling different organisms of the food web, and also evaluate the importance of local sources for these compounds by contrasting a pristine lake assumed only to receive contamination through LRAT, against a lake in an urban area with known local sources of contaminants like PCBs, DDTs, PAHs (polycyclic aromatic hydrocarbons) and cVMS (cyclic volatile methyl siloxanes) (Christensen, 2009; Krogseth et al., 2017a).

## 2 Materials and methods

### 2.1 Study area

**FIGURE 1:**
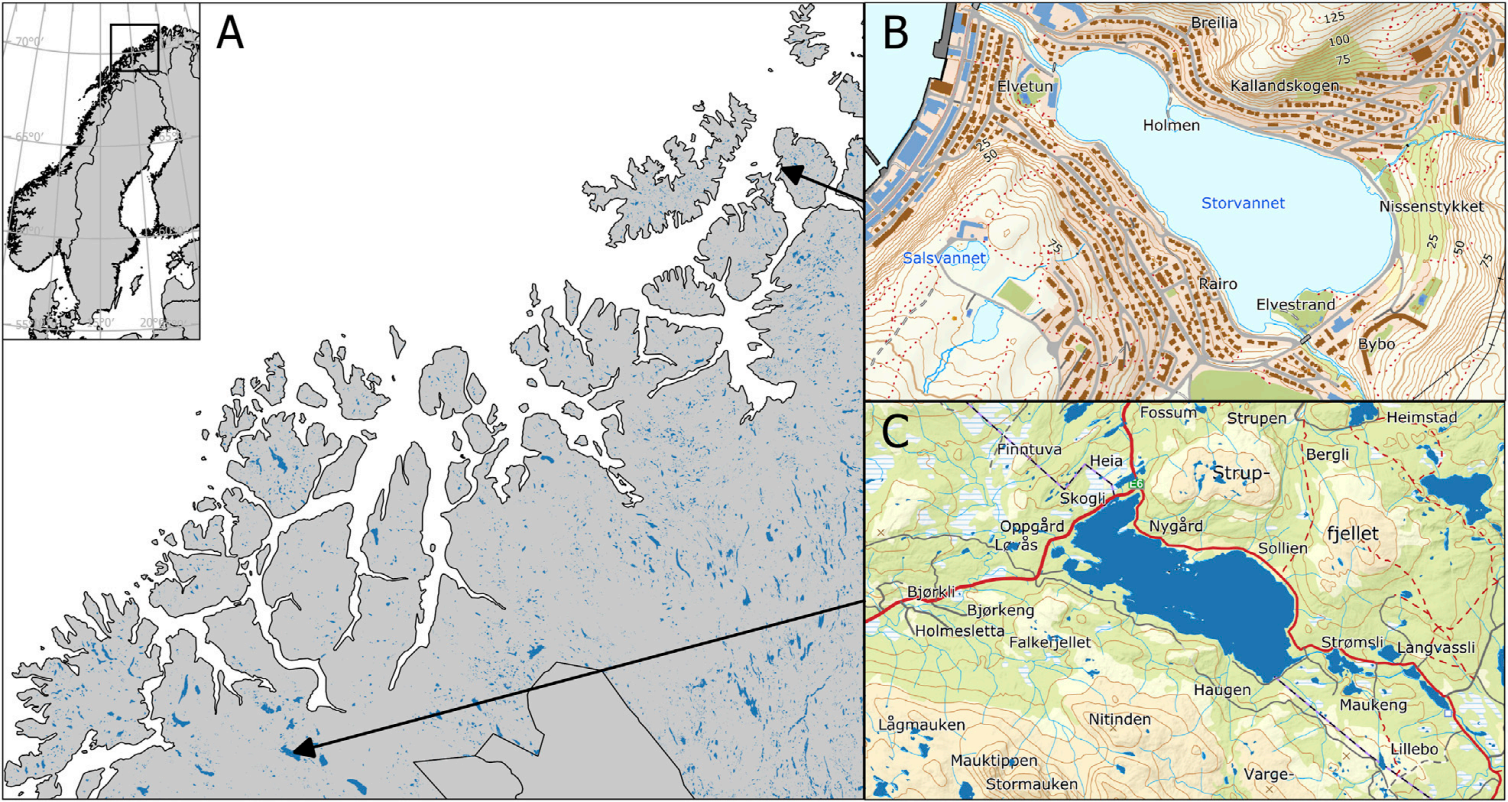
Map of Northern Norway **(A)** showing the location of Storvannet **(B)** and Takvannet **(C)**. **(B)** is based on data from the Norwegian Lake Database, available from The Norwegian Water Resources and Energy Directorate (NVE). Map data in **(A, C)** are from the Kartdata3 WMS service.

Two lakes in Northern Norway were selected for this study: Storvannet ( $70^{\circ }39^{\prime }$ N $23^{\circ }$ E) and Takvannet ( $69^{\circ }07^{\prime }$ N $19^{\circ }05^{\prime }$ E) (Figure 1). Both lakes are oligotrophic, have similar food webs, same top predators and have been well described and studied (Rikardsen and Elliott, 2000; Rikardsen et al., 2000; Amundsen et al., 2007; Amundsen et al., 2009; Amundsen et al., 2019; Eloranta et al., 2013; Eloranta et al., 2015; Krogseth et al., 2017a; Krogseth et al., 2017b). However, they are assumed to have different sources of contamination. Storvannet is in a residential area of Hammerfest city (population approx. 8000) and has a long history of contamination with legacy POPs from local sources (Christensen et al., 2009; Krogseth et al., 2017a; Krogseth et al., 2017b). The lake has an area of 0.23 km^2^, with an average depth of 9 m, maximum depth of 18 m, and a water renewal time of ca. 9 days in summer and 38 days in winter (Rikardsen et al., 1997). The lake is covered with ice from November until late May/early June (Krogseth et al., 2017b). Water temperatures through the whole water column are close to zero during winter (0°–1°C) and increase rapidly in June to a maximum of ca. 12°C in July (Rikardsen et al., 2000). Takvannet is located at 214 m above sea level with no known local sources of pollution. It has an area of 15 km^2^ with a maximum depth of 80 m. The lake is usually ice covered from late November to early June. During wintertime, the littoral temperature is close to zero while the profundal waters reach 2°C and during summer a maximum temperature of 12°–14°C can be reached (Klemetsen et al., 1989; Amundsen et al., 2009; Prati et al., 2020; Prati et al., 2021).

### 2.2 Sample collection

Samples were collected in Storvannet and Takvannet during September and October 2018, respectively. In both lakes, brown trout (*Salmo trutta*), Arctic char (*Salvelinus alpinus*), three-spined sticklebacks (*Gasterosteus aculeatus*), benthic invertebrates and sediments were sampled. Trout and char were caught with multinets (40 m long and 1.5 m high with a mesh size 10, 12.5, 15, 18,5, 22, 26, 35, 45 mm (knot to knot) (Supplementary Table SI.1). Sticklebacks were sampled either by deploying traps baited with shrimp wrapped in fine mesh (so the fish could not eat it) or by electrofishing in the shallow areas of the lakes. Fish were identified and their fork length and body weight measured. Otoliths were removed for age determination in both char and trout. Samples were individually wrapped in double aluminum foil and stored in zip-lock bags. Benthic organisms were collected from sieving sediment obtained with an Ekman grab (area 225 cm^2^). Organisms were sorted to taxa and stored in glass jars. Sediments were taken with an Ekman grab (area 225 cm^2^) at 5 different locations in TA and 4 at ST, at water depths between 12 and 16 m in Storvannet and between 18 and 25 m in Takvannet. The top 2 cm of each sediment sample was transferred to glass jars. All samples were stored at −20°C until further sample preparation in the lab.

### 2.3 Quality control procedures

All dissection and laboratory procedures were performed in an ISO class 6 clean room with both gas and particle filtration to limit the risk of contamination of samples. Detailed quality control procedures are described in SI. Samples were extracted by batches corresponding to a given lake and type of sample (trout, char, sticklebacks, benthic organisms or sediments) to avoid cross-contamination. Three laboratory blanks consisting of sodium sulphate were analyzed for each batch of samples, and one SRM (sample reference material) was analyzed for each sample type. Samples were spiked with isotopic labeled internal standards prior to extraction, and a recovery standard was added just before instrumental analysis (Detail information can be found in the SI).

### 2.4 Sample preparation

#### 2.4.1 Fish and benthic organisms

Extraction and clean-up of tissue samples was performed as described by Herzke et al. (2009) and Bouwman et al. (2008). For trout and char, 2 g of muscle were homogenized by finely chopping the tissue with a scalpel in a petri dish. For sticklebacks, 3 g of whole fish (pooled from 3 to 4 fish of similar size) were homogenized, while for benthos 3–5 g of whole organisms of each taxa were homogenized. Homogenized tissues were then mixed with 1:10 parts of sodium sulfate until they formed a fine powder and left in a freezer (−20°C) overnight. Internal recovery standards (see SI for more details) were added to each sample, before they were extracted for 15 min three times using cyclohexane/acetone (3:1) in an ultrasonic bath. Extracts were transferred to 100 mL glass vials and evaporated to 0.5 mL using a RapidVap (Labconco™ RapidVap™ Vacuum Evaporation System) and then left for evaporation to dryness under loose foil in the cleanroom. Lipid content was determined gravimetrically (Herzke et al., 2009). Samples were reconstituted with 1 mL of n-hexane and transferred to a glass centrifuge tube. Acid clean-up was performed by adding approximately 4–5 mL of concentrated H_2_SO_4_ to the sample followed by vortexing. Samples were left overnight in darkness. The following day, samples were centrifuged for 20 min at 2000 rpm. The hexane layer was then transferred to a new glass centrifuge tube, new acid was added, and samples were left for 1 h in darkness. This procedure was repeated 3 times. Further clean-up was performed using a 250 mm Pasteur pipette packed with sodium sulphate and preconditioned with 4 mL of n-hexane. The sample was eluted with 4 mL of n-hexane. The eluate was reduced to 0.5 mL using RapidVap and the solvent changed to isooctane. Samples were then evaporated to a final volume of 0.2 mL and transferred to GC-vials, to which recovery standards were added.

#### 2.4.2 Sediments

Sediment samples were thawed and spread out in aluminum trays and left to dry in the clean room for 48 h. Extraction of sediments was performed on an ASE Dionex Accelerated Solvent Extraction system (Thermo Fisher Scientific) equipped with 34 mm stainless steel extraction cells and 80 mL collection bottles. The extraction was performed with acetone:hexane (1:1 v/v) solvent at 150°C and 1500Psi. The packing of the cell was as follows: first a celluloce filter was placed into the cell outlet, followed by 5.0 g of silica, another filter, and a mixture of 5 g of dried sediments mixed with diatomaceous earth. All samples were spiked with internal standards before extraction. Extracts were transferred to 100 mL glass vials and evaporated with RapidVap to 1 mL and acid clean-up was performed as described above for fish and benthic organisms. Once the extract was clean, activated copper (−40 + 100 mesh, 99.5%) was added to remove all traces of sulfur. A second clean-up procedure was performed with Silica columns, previously rinsed with 10% dichloromethane in hexane. Samples were eluted with 40 mL of the same solvent and collected in a 100 mL glass vial. Samples were then evaporated to approximately 10 mL before transferring them to 15 mL vials and further reduced to 0.5 mL and then solvent changed to isooctane. Samples were then evaporated to a final 0.2 mL and transferred to GC vials.

#### 2.4.3 Total organic carbon and stable isotopes

Total organic carbon (TOC) was measured in sediment samples following the method DIN19539:2016-12. Biota and sediments from both lakes were analyzed for stable isotopes of carbon and nitrogen, and isotopic ratios were calculated (δ^13^C and δ^15^N). Detailed description for both methodologies can be found in the SI. The isotopic ratio of nitrogen (δ^15^N) was used to investigate the bioaccumulation of the chemicals in the two lakes, by regressing chemical concentrations against δ^15^N.

### 2.5 Instrumental analyses and quantification

#### 2.5.1 PCBs

PCBs were analyzed on an Agilent Technology 7890 GC and detection on an Agilent Technology 5975 MSD. For more detailed information, please refer to (Herzke et al., 2009).

#### 2.5.2 Dechloranes and chlorinated paraffins

Dechlorane analysis was performed on a Thermo Scientific Q Exactive Orbitrap GC/HRAM mass spectrometer (Thermo Fisher Scientific, Waltham, MA United States) with 1uL injections into a PTV (program temperature vaporizer) with a deactivated baffled glass liner at 70^°^C and after 0.1 min ramped to 320^°^C at 12^°^C/sec and held for 6 min. Helium was used as carrier gas at 1.4 mL/min in constant flow mode. A Thermo Scientific TG-5SilMS capillary column (15 m x 0.25 mmID, 0.25 um df) was used. The GC oven was programmed from 60^°^C (2 min) and ramped at 50^°^C/min to 320^°^C and held for 6 min with a total runtime of 13.2 min. Transfer line of MS was held at 300^°^C while ion source was set at 180^°^C in NCI mode with methane at 1.4 mL/min at 70eV with a mass resolution of 60,000 @ m/z 200, scanning at 250–700 m/z. The dechlorane standard was injected as a single point calibration. Results were processed in Tracefinder 4.1.

#### 2.5.3 Quantification of chlorinated paraffins

The CP calibration standards were prepared in isooctane from the stock solutions and ranged from 0.5 ng/μL to 5 ng/μL with observed linear relationships of *R* ^2^ = 0.99 (ΣS/MCCPs). The pattern deconvolution approach was used to quantify the CPs (Bogdal et al., 2015). A detailed description of the statistical analysis was reported by Al Saify et al. (2021), using the same commercially available technical formulations with two additional single-chain length standards (C_11_ 60% and C_12_ 70% Cl) to reconstruct the CP sample patterns.

### 2.6 Data analyses

Data are presented on both lipid normalized (lw) and wet weight (ww) basis for biota and normalized for total organic carbon (oc) and dry weight (dw) for sediments. However, statistical analyses were performed on lw and dw. Due to the overall small sample sizes and associated likely deviation from normal distributions, data were summarized using median and range. The median is much less affected by extreme observations in either the upper or lower tails of the distribution (Huston and Juarez-Colunga, 2009) and is therefore often chosen as a more “robust” and better measure of center than the more commonly used mean.

We used the Wilcoxon rank sum test to test differences between lakes for all the morphometrics, lipid percent, isotopes and TOC.

To test for differences in contaminant mass fraction between lakes and compartments, we performed an Aligned Rank Transform two-way factorial ANOVA (Wobbrock et al., 2011), using the ARTool package for R (Elkin et al., 2021). This is a non-parametric equivalent of a normal two-way factorial ANOVA, appropriate for data that cannot be assumed to conform to the requirements for traditional parametric methods. We performed a contrast analysis on the estimated marginal means (a.k.a. The least squares means) from the ART analysis, across the reference grid of predictor variables, using functions available in the emmeans package for R (Russell, 2020). For PCBs the analysis was performed on the ∑7PCBs (28, 52, 101, 118, 138, 153, 180) which is often used for comparison of mass fractions over time or between different locations unless specified otherwise. It is important to highlight that caution should be made in interpreting results due to low sampling numbers, especially for benthic species. For Statistical analyses, values below limit of detection (LOD) were substituted with a value of LOD/2 (Giskeødegård and Lydersen, 2022).

Biota sediment accumulation factor (BSAF) can be used as an approach to estimate the bioaccumulation potential of organic contaminants. BSAFs were calculated as the lipid normalized concentration in biota divided by the mean of the OC- normalized concentration in sediments in the lake (Burkhard et al., 2004) for samples that had values above LOD.

All statistical analyses were performed in R (R Core Team, 2020).

## 3 Results and discussion

### 3.1 Morphometrics and stable isotopes

Summary statistics for organism morphometrics (length, weight and age for char and trout), total organic carbon (TOC) in sediments, stable isotopes, and lipid content for samples from both lakes are shown in Supplementary Tables SI.1, SI.2. There were no significant differences in length, weight, age and lipid content between the two lakes for either trout (Wilcoxon rank sum test length: W_x_ = 58.5, *p* = 0.83, weight: W = 59, *p* = 0.81, age: W = 70, *p* = 0.3, lipid: W = 53, *p* = 0.92) or char (length: W = 63, *p* = 0.15, weight: W = 60, *p* = 0.24, age: W = 59, *p* = 0.26, lipid: W = 42, *p* = 0.84). Surface sediment samples from Storvannet had significantly higher proportions of TOC compared to Takvannet (4.3%–6.5% vs 2.0%–2.3%, W = 20, *p* = 0.015).

Supplementary Figure SI.1 presents the stable isotope ratios in both lakes. δ ^15^N ratios were significantly higher in Storvannet than in Takvannet for both char (W = 82, *p* < 0.001) and trout (W = 101, *p* < 0.001), while no significant differences were observed between lakes for sediments, benthic organisms, or sticklebacks. Within lakes there were no significant differences between char and trout. However, a few trout individuals had higher δ ^15^N ratios than char in both lakes (Supplementary Figure SI.1). Trout is considered to be competitively superior to char and it has been observed that the main prey of trout over 300 mm in length is three-spined sticklebacks but they can also become cannibalistic (Prati et al., 2021). Char and trout are omnivorous and feed on different prey depending on their ontogenetic development (Klemetsen et al., 2003) but may also be influenced by the environmental conditions of the lakes and interspecific competition which can vary between lakes (Guildford et al., 2008; Cabrerizo et al., 2018; Prati et al., 2021). Although there were no statistically differences in length within or between lakes, the fish over 300 mm had higher δ ^15^N values and were more abundant in the samples from Storvannet. The differences between the fish from the two lakes may indicate that fish in Storvannet rely to a greater extent on fish prey compared to fish in Takvannet. In addition, these differences may be explained by potentially higher microbial activity in Storvannet. The microbial loop has been shown to play an important role in the uptake of terrestrial material into the food-web (Poste et al., 2019) and can also add additional trophic levels (Karlsson et al., 2012; Mc Govern et al., 2019).

Values of δ ^13^C were not significantly different between lakes for char, trout, stickleback, and benthos, but were significantly enriched in sediments from Takvannet compared to Storvannet. This may indicate a higher terrestrial input into Takvannet, which has a larger catchment area (59.2 km^2^) than Storvannet (42.4 km^2^) (Norwegian Water Resources and Energy Directorate (NVE), 2020). This is coupled with the shorter water turnover time in Storvannet due to its large drainage basin and small size.

### 3.2 PCBs

Overviews of ∑7-PCB and PCB-153 mass fractions in sediments and biota from both lakes are provided in Table 1, Supplementary Table SI.3, and Supplementary Figures SI.2A, B. Mass fractions in sediment were assessed according to the guidelines given by the Norwegian Environment Agency (Miljødirektoratet, 2016) to obtain information on the environmental condition of the lakes. Sediment mass fraction of ∑7-PCB in Storvannet ranged from 21.4 to 65.9 ng/g dw, with a median of 43.9 ng/g dw. This is comparable to those measured in 2006 (range of 54.9–58.4 ng/g dw, Evenset et al., 2006), and corresponds to condition class III (moderately polluted) (Miljødirektoratet, 2016). Storvannet is more polluted than other lakes both on mainland Norway (average ∑7-PCB 1.9 ng/g dw, *n* = 49) and on Svalbard (average ∑7-PCB 10.1 ng/g dw, *n* = 5) (Gabrielsen et al., 2011). This, together with its classification as “moderately polluted” corroborates that it receives PCBs from local sources (Christensen, 2009; Krogseth et al., 2017a; Krogseth et al., 2017b). Mass fractions of ∑7-PCB in Takvannet sediments were significantly lower than those in Storvannet (W = 20, *p* = 0.01), ranging from 0.14 to 0.49 ng/g dw. Mass fractions in Takvannet were also significantly lower than those from Storvannet when normalized to TOC content, despite lower TOC content in Takvannet sediments. The ∑7-PCB in Takvannet correspond to background conditions (Miljødirektoratet, 2016). This suggests that Takvannet mainly receives PCBs through LRAT.

**TABLE 1:** Mass fractions of ∑7-PCB and of PCB-153 in sediments (ng/g dw) and biota (ng/g lw) in Storvannet and Takvannet.

		∑ PCB 7		PCB 153	
Lake	n	Median	Range	Median	Range
Storvannet					
Sediment	4	44	21–66	11	6–18
Benthos	2	839	708–971	262	215–309
Stickleback	5	1121	1030–1357	371	342–455
Trout	11	633	55–3866	145	17–909
Char	10	1121	57–2401	262	15–638
Takvannet					
Sediment	5	0.21	0.1–0.5	0.04	0.03–0.1
Benthos	5	28	9–36	7	3–10
Stickleback	3	67	58–80	22	19–25
Trout	10	40	17–532	10	4–106
Char	9	145	83–1078	36	21–269

Consistent with this, ∑7-PCB mass fractions in biota were higher in Storvannet than in Takvannet for all organisms, except char. This was especially evident at lower trophic levels, where mass fractions could differ by as much as 100-fold, while differences become gradually smaller for species higher up the food chain. As a result, in Takvannet, mass fractions increase considerably with increasing δ^15^N, while in Storvannet mass fractions do not increase as sharply (Supplementary Figures SI.2A, B, SI.5). The correlation between PCB-153 and δ ^15^N differs between lakes. In Takvannet we observed a significant positive relationship (linear model of log_10_ (conc_PCB-153_) ∼ δ ^15^N; slope = 1.39, 95% CI: 1.191–1.561), while in Storvannet the correlation was slightly negative but not significant (slope = 0.96, 95% CI: 0.826–1.104) (Supplementary Figures SI.3, SI.5). The opposing relationship of PCB-153 and ∑PCB7 observed in Storvannet resembles the observed and modelled behavior of cyclic volatile methyl siloxanes (cVMS) in Storvannet (Krogseth et al., 2017b). This was explained by the particular characteristics of both the physical environment and food web of Storvannet. This lake has a rapid water turnover time, resulting in a disequilibrium situation between water and sediments (with higher fugacity in sediment than in water). This results in lower mass fractions in the benthic-feeding fish due to more efficient elimination of contaminants through ventilation to water (Krogseth et al., 2017a; Krogseth et al., 2017b). Hence, the lack of biomagnification of PCB-153 in Storvannet is likely a result of these processes. See the Supplementary Material for a more detailed discussion of the bioaccumulation behavior and BSAFs of PCBs in these two lakes. Supplementary Figure SI.5 shows the results of a principal component analysis (PCA) highlighting differences between compartments and lakes. The first two principal components (PC) explained 66.8% of the variance in the data (PC 1: 41.9%, PC 2: 24.9%). The first PC mainly accounts for differences between lakes while the second PC is related to differences between compartments.

### 3.3 Dechloranes

#### 3.3.1 Dechlorane Plus

Mass fractions of *syn*- and *anti*-DP in sediments and biota of Storvannet and Takvannet are provided in Table 2 and Supplementary Table SI.4. Both DP isomers were detected in all sediment samples. Mass fractions (in ng/g dw and ng/g OC) were higher in Storvannet (*syn*-DP median: 9.56 ng/g OC range: 3.50–38.0 ng/g OC, *n* = 4; *anti*-DP median: 43.6 ng/g OC; range: 9.16–153 ng/g OC, *n* = 4), than in Takvannet (*syn*-DP median: 0.26 ng/g OC, range: 0.08–0.41 ng/g OC, *n* = 5; *anti*-DP median: 0.86 ng/g OC; range: 0.44–3.72 ng/g OC, *n* = 5), with consistently higher *anti*-DP than *syn*-DP mass fractions in both lakes. Higher mass fractions of ∑DP in Storvannet than in Takvannet indicate that Storvannet also receives DP from local sources around the lake, as it does for other organic contaminants including PCBs and cVMS (Krogseth et al., 2017a; b; Christensen et al., 2009; Christensen, 2009). Mass fraction ranges of the sum of anti-DP and syn -DP (∑DP) in sediment samples from Storvannet were comparable to those found in Lake Erie (0.061–8.62 ng/g dw) (Sverko et al., 2008), but higher than in Lake Huron and Lake Michigan (∑DP 0.87 ng/g dw and 0.55 ng/g dw, respectively) (Shen et al., 2011b). However, in Storvannet, mass fractions were lower than in Lake Ontario (*syn*-DP median: 30 and 35 ng/g dw; *anti*-DP median: 115 and 176 ng/g dw, respectively) (Qiu et al., 2007; Tomy et al., 2007), which has elevated ∑DP mass fractions because of its location downstream of a DP manufacturing plant (Shen et al., 2011a). Ranges of ∑DP in sediment samples from Takvannet were comparable to those found in Lake Winnipeg of ∑DP = 0.03 ng/g; *syn*-DP: 0.011 ng/g dw, *anti*-DP: 0.018 ng/g dw. Takvannet is considered a remote lake, and the low mass fractions suggest that it only receives DP through LRAT as it was seen in Lake Winnipeg (Qiu et al., 2007; Tomy et al., 2007).

**TABLE 2:** Mass fractions of syn-DP, anti-DP, and DP total (syn-DP + anti-DP) measured in sediment (ng/g OC) and biota (ng/g lw) in Storvannet and Takvannet, as well as the calculated f_anti_ fraction. The numbers in parenthesis indicate the number of samples > LOD. Mass fractions < LOD are not included in medians and ranges. “na” indicates that the fanti fraction and DP total concentration was not calculated because none of the DP isomers were detected > LOD.

		syn-DP			anti-DP			F*anti*			DP total	
Lake	n	Median	Range	n	Median	Range	n	Median	Range	n	Median	Range
Storvannet												
Sediment	4 (4)	9.56	3.50–38.0	4 (4)	43.6	9.16–153	4 (4)	0.82	0.78–0.85	4 (4)	3.23	1.24–12.5
Mollusc	1 (1)	0.67	-	1 (1)	2.49	-	1 (1)	0.79	-	1 (1)	3.15	-
Chironomid	1 (1)	1.16	-	1 (1)	3.26	-	1 (1)	0.74	-	1 (1)	4.42	-
Stickleback	5 (5)	0.10	0.08–0.15	5 (5)	0.18	0.13–0.28	5 (5)	0.65	0.63–0.66	5 (5)	0.28	-
Arctic char	10 (1)	0.40	-	10 (2)	0.56	0.53–0.60	10 (1)	0.6	-	10 (2)	0.77	0.53–1.00
Brown trout	10 (0)	< LOD	-	10 (2)	0.19	0.15–0.23	10 (0)	na	-	10 (2)	0.19	0.15–0.23
Takvannet												
Sediment	5 (5)	0.26	0.08–0.41	5 (5)	0.86	0.44–3.72	5 (5)	0.80	0.77–0.91	5 (5)	0.03	0.01–0.14
Amphipods	1 (0)	< LOD	-	1 (1)	0.07	-	1 (0)	na	-	1 (1)	0.07	-
Valvatidae	1 (1)	0.20	-	1 (1)	0.61	-	1 (1)	0.76	-	1 (1)	0.80	-
Lymnae	1 (1)	0.30	-	1 (1)	1.90	-	1 (1)	0.86	-	1 (1)	2.22	-
Mollusc	1 (1)	0.77	-	1 (1)	0.58	-	1 (1)	0.43	-	1 (1)	1.35	-
Chironomid	1 (1)	0.17	-	1 (1)	0.20	-	1 (1)	0.54	-	1 (1)	0.37	-
Stickleback	3 (1)	0.05	-	3 (3)	0.03	0.03–0.09	3 (1)	0.64	-	3 (3)	0.03	0.03–0.14
Arctic char	9 (0)	< LOD	-	9 (0)	< LOD	-	9 (0)	na	-	9 (0)	na	-
Brown trout	10 (0)	< LOD	-	10 (0)	< LOD	-	10 (0)	na	-	10 (0)	na	-

**FIGURE 2:**
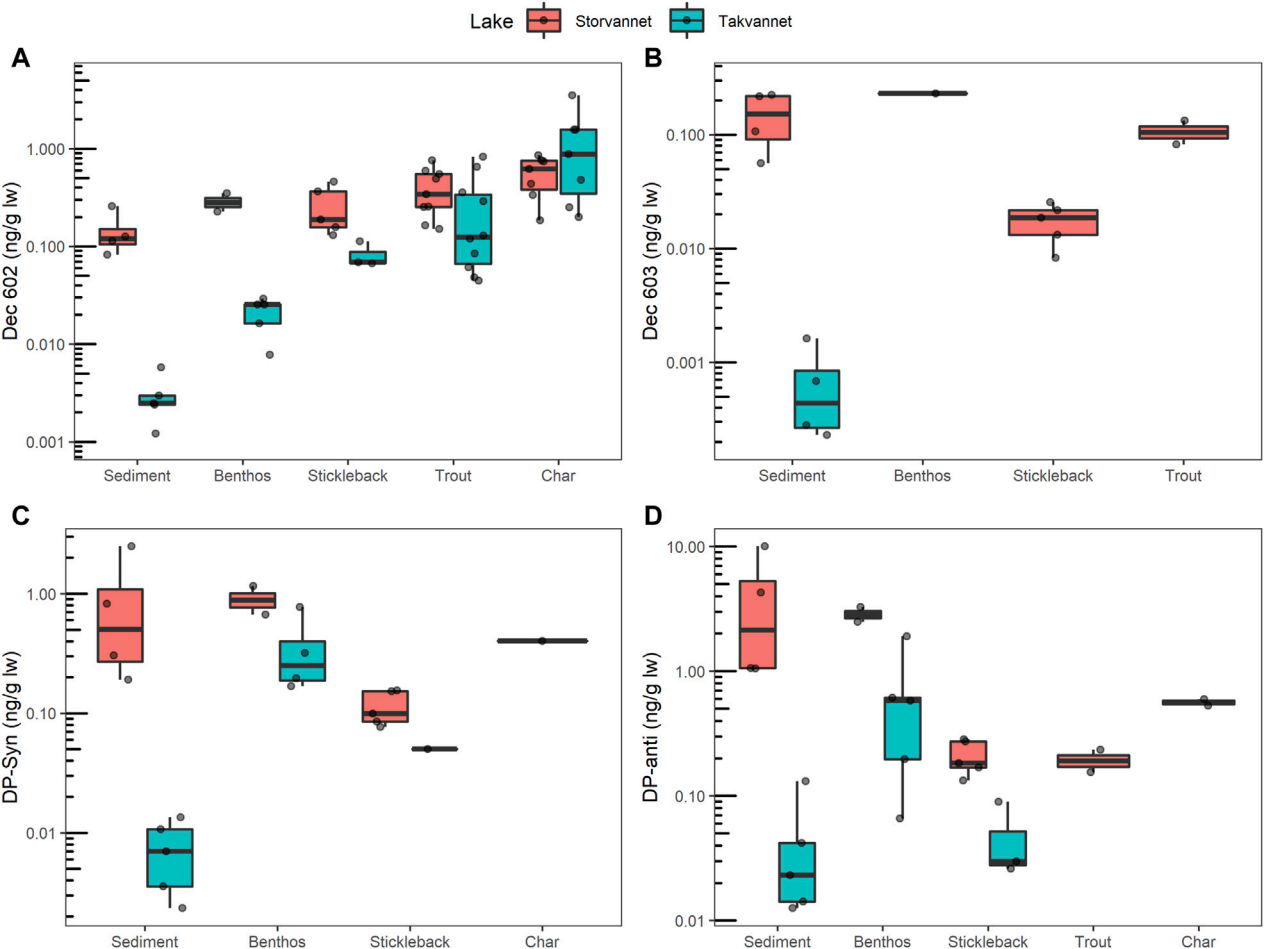
Mass fractions of **(A)** Dec 602, **(B)** Dec 603, **(C)** syn-DP and **(D)** anti-DP in sediment and biota from Storvannet and Takvannet. Dots represent each sample and black lines inside the boxes represent the median. The box covers the 25th to 75th percentile of the data. The upper whisker extends from the hinge to the largest value no further than 1.5 * IQR from the hinge (where IQR is the interquartile range, or distance between the first and third quartiles). The lower whisker extends from the hinge to the smallest value at most 1.5 * IQR of the hinge. Data beyond the end of the whiskers are “outlying” points and are plotted individually. Values below LOD are not included, thus char and trout are not included in **(B, C)**, respectively.

In biota from Storvannet, *syn*- and *anti*-DP were above detection limits in all samples of benthos and sticklebacks. In biota from Takvannet, syn- and anti-DP were above detection limits in most samples of benthos and sticklebacks, but not in any of the char or trout. Like for sediments, mass fractions of both isomers in biota were significantly higher in Storvannet than in Takvannet (Figure 2), and *anti*-DP were consistently higher than *syn*-DP mass fractions (Table 2 and Supplementary Table SI.4). S*yn*-DP and *anti*-DP in benthos ranged from 0.67 to 1.16 ng/g lw (*n* = 2) and 2.49–3.26 ng/g lw (*n* = 2), respectively, in Storvannet. In Takvannet, amphipods were the only benthic sample with mass fractions of *syn*-DP below LOD. The rest of the benthic samples had *syn*-DP that ranged from 0.17–0.77 ng/g lw (*n* = 4) and mass fractions of *anti*-DP that ranged from 0.07–1.90 ng/g lw (*n* = 5) with the lowest mass fraction found in amphipods.

In Storvannet, mass fractions of *syn*-DP and *anti*-DP in sticklebacks ranged from 0.08–0.15 ng/g lw (*n* = 5) and 0.13–0.28 ng/g lw (*n* = 5), respectively. In Takvannet, only one stickleback out of three had mass fractions above LOD (0.05 ng/g lw) of *syn*-DP. In contrast, mass fractions for *anti*-DP were measured in all three-spined stickleback with ranges of 0.03–0.09 ng/g lw (*n* = 3). In both lakes there was a decrease in mass fractions from benthic organisms to sticklebacks for both isomers.

In Storvannet, *syn*-DP was detected in one char (0.40 ng/g lw) and *anti*-DP was found in two char samples (0.53–0.60 ng/g lw) and two trout samples (0.15–0.23 ng/g lw). The detected mass fractions were comparable to, or higher than, the mass fractions measured in sticklebacks from the same lake. Our results are similar to other studies, where DPs were detected in low mass fractions in fish despite elevated mass fractions in sediments (Guo et al., 2017). In Southern Norway, 95% of DP mass fractions in fish samples were below the limit of quantification in the urban lake Mjøsa and in the pristine lake Femunden with detected ∑DP values ranging between 0.05–0.10 ng/g ww. (Jartun et al., 2020). The few detected *syn-*DP and a*nti-*DP mass fractions in Storvannet char were slightly higher than in Trout or Walleye from all of the Great Lakes, except for *anti*-DP in Lake Huron which were higher than in Storvannet (Guo et al., 2017). Moreover, they were also comparable to those in Arctic char from Greenland (Vorkamp et al., 2019b), where *syn-*DP was not detected, and anti-DP was detected in low mass fractions in 3 out of 6 samples (0.047–0.188 ng/g lw). The trout samples from Storvannet had *anti-DP* mass fractions that were only slightly higher than those from Lakes Michigan and Superior (Guo et al., 2017). Other studies of trout from the Great Lakes have found higher ∑DP mass fractions with a mean of 0.35 ng/g lw (Kurt-Karakus et al., 2019) and also in a range from 2.3–7.2 ng/g lw (Lake Ontario only) with the highest mass fractions from 1988 (Ismail et al., 2009). In Takvannet neither of the DP isomers were found in char or trout. Supplementary Figure SI.7 shows the PCA biplot for DPs and DP related compounds. The plot highlights a clear separation between compartments and to a lower degree between lakes. The low detection frequency of *syn*-DP and *anti*-DP in char and trout species in both Storvannet and Takvannet compared with levels in samples from benthic organisms or stickleback samples in the same lake, indicate a lack of magnification for DP isomers in both lakes. BSAF for both anti and syn DP (Supplementary Figure SI.8) suggest low bioaccumulation potential. In Takvannet, BSAF could only be estimated for benthic organisms (average 1.11 and 0.46 for syn and anti DP) and sticklebacks (average of 0.03 for syn DP and 0.06 for anti DP). In Storvannet, BSAF values were lower than 1 in all organisms, including benthos (0.04 and 0.06 for anti and syn DP), sticklebacks (0.003 and 0.01 for anti and syn DP) and char (0.002 and 0.003 for anti and syn DP). The low BSAF values of both DPs in both lakes indicate a lack of biomagnification. However, these BSAFs should be interpreted with care due to the small sample size. Similar results were observed but with trophic magnification factors (TMFs) in Lake Ontario by Tomy et al. (2007), where low TMF values for *syn*- and *anti-*DP indicated a lack of biomagnification. Results from Lake Winnipeg were more ambiguous, with an estimated TMF of 2.5 for *anti*-DP, but a lack of biomagnification for *syn*-DP (Tomy et al., 2007). In contrast, Wu et al. (2010) found TMFs of 6.5 and 11.3 for *anti*-DP and *syn-*DP, respectively. It has been suggested that the lack of increasing DP with increasing trophic level may be explained by DPs low water solubility and high octanol water partition coefficient (log *K*_ow_ = 11.3, Shen et al., 2011b). DPs high hydrophobicity means that it binds to organic carbon both in sediments and in the water column and hence is less bioavailable. The high log *K*_OW_ limits the transfer efficiency across the gastrointestinal tract, hence decreasing the organism’s uptake of DP from the diet (Hoh et al., 2006; Guo et al., 2017). It is also possible that DPs become biotransformed by fish, as has been described by Hoh et al. (2006). Previous studies have concluded that DP bioaccumulation patterns are species-specific rather than simply correlated with general trophic level, and that differences in biomagnification also can be related to differences in ecosystem structure (Tomy et al., 2007; Vorkamp et al., 2015; Schlabach et al., 2017; Vorkamp et al., 2019b). Biomagnification of DP isomers has been detected in freshwater food webs (Tomy et al., 2007; Wu et al., 2010) but also in marine food webs (Peng et al., 2014; Na et al., 2017; Zhang and Kelly, 2018). The reasons why they display biomagnification in certain species/food-webs, but not in others, is not clearly known yet.

#### 3.3.2 DP stereoisomer profiles

The commercial DP products consist of around 65% *anti*-DP and 35% *syn*-DP, giving an $f_{anti}$ value of 0.65. However, this can range between 0.59 and 0.80 due to differences in production batches and manufacturers (Hoh et al., 2006). Isomer (*f*_*anti*_) ratios found in the environment that deviate from this may show the extent of environmental degradation and/or biotransformation of the different isomers as the two stereoisomers may degrade at different rates (Xian et al., 2011). The median *f*_*anti*_ values in sediments from Storvannet and Takvannet were 0.82 (range 0.78–0.85) and 0.80 (range 0.77–0.91), respectively (Table 2). These are at the high end of those reported for technical mixtures (Hoh et al., 2006; Wang et al., 2010), suggesting a depletion of the *syn*-DP isomer. This agrees with observations in sediments from Lake Ontario (*f*_*anti*_ = 0.86) (Tomy et al., 2007). It also agrees with higher *f*_*anti*_ in sediment samples closer to source regions (Hoh et al., 2006).

The *f*_*anti*_ values in benthos were higher in Storvannet than in Takvannet for both mollusks (ST: *f*_*anti*_ = 0.79, TA: *f*_*anti*_ = 0.43) and chironomids (ST: *f*_*anti*_ = 0.74, TA: *f*_*anti*_ = 0.54). This indicates a depletion of *anti*-DP in benthos from Takvannet compared to in Storvannet. It also indicates a depletion of *anti*-DP in benthos compared to sediments in Takvannet. However, when all benthic samples in Takvannet were considered, the range in *f*_*anti*_ was large (0.43–0.86), and it is hard to draw conclusions due to a low number of samples. The median *f*_*anti*_ values in sticklebacks were not different between the two lakes (ST: 0.65, TA: 0.64) and were within the range observed for commercial DP products.

Due to low detection frequencies of DP in trout and char, *f*_*anti*_ could only be estimated for one char sample from Storvannet (0.60). This value is in the same range as *f*_*anti*_ in fish from Lake Ontario (0.65 ± 0.06) (Tomy et al., 2007), Lake Superior (0.63 ± 0.07) and Lake Erie (0.60 ± 0.07) (Guo et al., 2017). As in other studies (Tomy et al., 2007; Shen et al., 2011a), we observed lower *f*_*anti*_ values for benthic organisms and fish than in sediments. In the food web from Lake Ontario, the values declined from 0.65 in plankton to 0.51 in trout (Tomy et al., 2007). This might be explained by a stereoselective accumulation of *syn*-DP, since *anti-*DP is more reactive and susceptible to photodegradation and biodegradation (Möller et al., 2010; Wang et al., 2015). Experiments have shown that the *syn* isomer had higher assimilation and lower depuration in rainbow trout than the *anti-*isomer (Tomy et al., 2008). It is not known if this happens in other organisms to the same extent (Wang et al., 2016).

#### 3.3.3 Other dechloranes

Dec 601 and 604 were below LOD in all samples in both Storvannet and Takvannet. This is in agreement with findings from the limited number of studies describing the presence of DP related compounds in freshwater ecosystems (Shen et al., 2010; Shen et al., 2011a; 2011b; Jartun et al., 2020). Neither Dec 601 nor 604 were detected in sediments or fish from Lake Mjøsa or Lake Femunden (Jartun et al., 2020), and were also not detected in sediments, fish or birds from the Oslofjord (Ruus et al., 2019). While Dec 604 has been described in sediments and fish samples from the Great Lakes (Sverko et al., 2011) it was only present in the sediment samples from Michigan, Huron, Erie and Ontario lakes and only in four samples from Lake Superior (Shen et al., 2010; Shen et al., 2011a), while in fish samples it was only present in trout from Lake Ontario (Shen et al., 2010). This suggests that the manufacturing plants along the Niagara River were the main sources of the detected Dec 604. Thus, it is not surprising that we did not detect Dec 604 in Storvannet and Takvannet. Moreover, this also agrees with a spatial study of Dechlorane Plus and related compounds in European background air, including Norwegian sites, where Dec 601 and 604 were not detected in air samples (Skogeng et al., 2023).

Dec 603 (Table 3; Supplementary Table SI.5; Figure 2) was detected in all sediment samples from both lakes (ST: 2.54 p/g OC, range: 1.28–3.95 pg/g OC; TA: 0.014 pg/g OC, range: 0.01–0.05 pg/g OC), and in the chironomid sample (0.23 ng/g lw, *n* = 1), all sticklebacks (0.02 ng/g lw, range: 0.01–0.03 ng/g lw, *n* = 5) and two trout samples (0.08–0.13 ng/g lw) in Storvannet. Dec 603 was not detected in biota from Takvannet. Similarly low Dec 603 mass fractions were found in sediment in the Oslofjord (average 0.069 ng/g dw (Ruus et al., 2019)) and in two studies in the Great Lakes (0.001–0.6 ng/g dw (Shen et al., 2010) and < LOD–1.1 ng/g dw (Shen et al., 2011b)). The Dec 603 mass fractions measured in Storvannet are in the low range in comparison to measurements in trout from Lake Superior, Huron and Ontario and in whitefish from Lake Erie, with mass fractions ranging from 0.014–0.500 ng/g lw (Shen et al., 2010). There is no clear current point source of Dec 603, thus this compound might enter the lakes mainly through atmospheric transport (Shen et al., 2010). However, Dec 603 was not measured above detection limits in air samples from background regions in Europe (Skogeng et al., 2023).

**TABLE 3:** Mass fractions of Dec 602 and 603 measured in sediment (pg/g OC) and biota (pg/g ww) in Storvannet and Takvannet. The numbers in parentheses indicate the number of samples > LOD. Mass fractions < LOD are not included in medians and range.

		Dec 602			Dec 603	
Lake	n	Median	Range	n	Median	Range
Storvannet						
Sediment	4 (4)	2.10	1.67–3.95	4 (4)	2.45	1.28–3.95
Mollusc	1 (1)	0.23	-	1 (0)	<LOD	-
Chironomid	1 (1)	0.35	-	1 (1)	0.23	-
Stickleback	5 (5)	0.19	0.13–0.46	5 (5)	0.02	0.01–0.03
Arctic char	10 (7)	0.62	0.18–0.86	10 (0)	<LOD	-
Brown trout	11 (8)	0.34	0.15–0.76	11 (2)	0.11	0.08–0.13
Takvannet						
Sediment	5 (5)	0.11	0.04–0.16	5 (5)	0.014	0.01–0.05
Amphipods	1 (1)	0.01	-	1 (0)	<LOD	-
Valvatidae	1 (1)	0.03	-	1 (0)	<LOD	-
Lymnae	1 (1)	0.03	-	1 (0)	<LOD	-
Mollusc	1 (1)	0.03	-	1 (0)	<LOD	-
Chironomid	1 (1)	0.02	-	1 (0)	<LOD	-
Stickleback	3 (3)	0.07	0.07–0.11	3 (0)	<LOD	-
Arctic char	9 (7)	0.88	0.20–3.51	9 (0)	<LOD	-
Brown trout	10 (10)	0.12	0.04–0.82	10 (0)	<LOD	-

Dec 602 was found in all sediment samples from both lakes, with mass fractions ranging between 1.67–3.95 pg/g OC in Storvannet (median 2.10 pg/g OC), and 0.04–0.16 pg/g OC (median 0.11 pg/g OC) in Takvannet (Figure 2; Table 3; Supplementary Table SI.5). Dec 602 was detected more frequently than Dec 603 in the biota samples. Dec 602 was detected in all benthos and stickleback samples, and in over 70% of trout and char samples, from both lakes. Dec 602 was higher in the single chironomid sample (0.35 ng/g lw) than in the single mollusk sample (0.23 ng/g lw) from Storvannet. Mass fractions in benthic organisms from Takvannet were lower than in Storvannet and ranged between 0.008–0.029 ng/g lw (*n* = 5). Similarly, Dec 602 in sticklebacks were higher in Storvannet (0.19 ng/g lw (range: 0.13–0.46, *n* = 5)) than in Takvannet (0.07 ng/g lw (range: 0.07–0.11, *n* = 3)) (Figure 2). Mass fractions in trout from Storvannet had a median of 0.34 ng/g lw (range: 0.15–0.76 ng/g lw, *n* = 9) while in Takvannet the median value was 0.12 ng/g lw (range: 0.04–0.82, *n* = 10). In contrast, char from Takvannet (0.88 ng/g lw, range 0.20–3.51, *n* = 7) had higher mass fractions than Storvannet char (0.62 ng/g lw, range: 0.18–0.86, *n* = 7). However, these differences between lakes for char and trout were not significant.

Takvannet had lower Dec 602 mass fractions in sediment than those reported for any of the Great Lakes (Shen et al., 2010), while Dec 602 in Storvannet sediment was higher in all of the Great Lakes except Lake Ontario h (0.160–11 ng/g dw) (Shen et al., 2010). Dec 602 median mass fractions in trout from Takvannet and Storvannet were 61 and 21 times lower, respectively, than those in trout from Lake Ontario (7.38 ng/g lw). Dec 602 mass fractions in char from Storvannet and Takvannet were lower than in char from all the Great Lakes, except Lake Superior.

**FIGURE 3:**
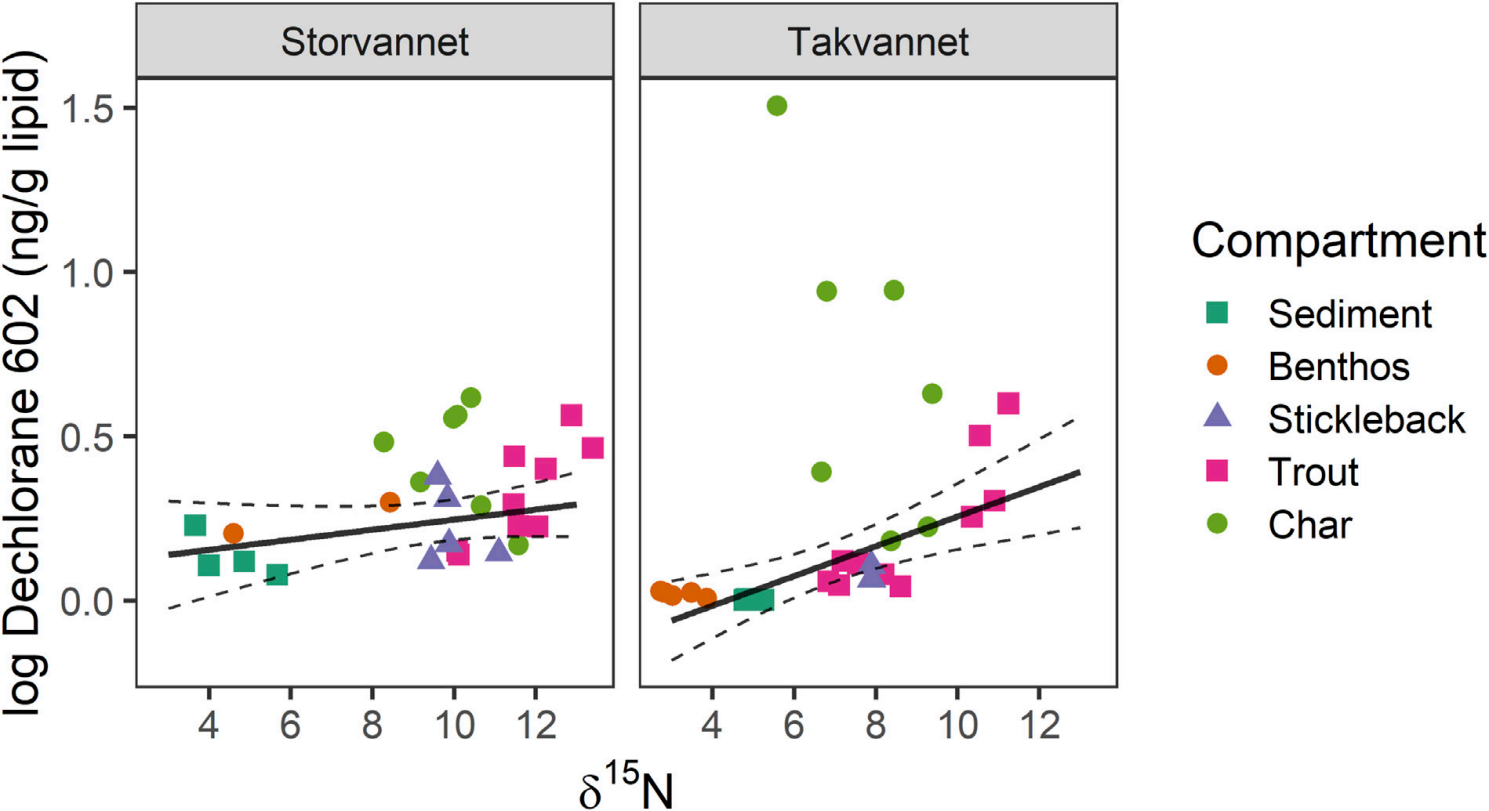
Relationship between δ ^15^N and the logarithmic Dec 602 mass fractions for all samples in Takvannet and Storvannet. Solid black line represents the fit of the robust linear model and the dash lines represent the 95% confidence interval of the fit.

While there were clear differences in sediment Dec 602 mass fractions between Storvannet and Takvannet, differences between lakes decreased with increasing δ ^15^N (Figure 2 and Supplementary Figure SI.9). This pattern was similar to the PCB behavior, suggesting that the lake characteristics in Storvannet play an important role in the biomagnification (or lack thereof) of pollutants in this lake (Krogseth et al., 2017a; Krogseth et al., 2017b). The linear model between Dec 602 and δ ^15^N showed a slightly positive correlation (slope: 1.01, 95% CI: 0.99–1.04) for Storvannet and (slope: 1.04, 95% CI: 1.02–1.05) for Takvannet (Figure 3). Increasing Dec 602 mass fractions with increasing δ ^15^N was significant in Takvannet which agrees with previous studies (Shen et al., 2011a; Feo et al., 2012; Barón et al., 2014a). It has been suggested that this chemical is more bioaccumulative and may have a higher bioavailability compared to Dechlorane Plus due to a log *K*_ow_ lower than 8.1 (Wang et al., 2016). BSAF values for Dec 602 (Supplementary Figure SI.8) show a clear bioaccumulation potential, particularly in Takvannet, while the values observed in Storvannet are low, which is in accordance with what we have previously described, and we attribute this to differences in lake characteristics. In Takvannet BSAF values increased from benthos (0.20) < sticklebacks (0.78) < trout (2.45) < char (11.3). In Storvannet values for benthos (0.12) were very similar to sticklebacks (0.11) followed by trout (0.14) and highest value of 0.23 for char.

The higher mass fractions of DP, Dec 602 and Dec 603 in Storvannet sediments compared to those in Takvannet suggest that Storvannet may receive not only LRAT input but also contribution from local emissions of these compounds. This is not unexpected, since DP and related compounds have been widely used as flame retardants in industrial applications and household products such as in electrical and electronic equipment (Wang et al., 2016). Production, use, recycling, waste handling, as well as leachate and run-off from landfills, wastewater treatment plants and more, can lead to their release (Wang et al., 2016; Schlabach et al., 2017; Wang and Kelly, 2017). High levels of DPs have been measured in sewage sludge and wastewater treatment plants from urban areas (de la Torre et al., 2012; Barón et al., 2014b; Schlabach et al., 2017), and they have also been found in dust and electronic appliances from residential areas (Newton et al., 2015; Schlabach et al., 2017; Wong et al., 2018; Sørensen and Larsen, 2019). Storvannet has received both intentional and unintentional emissions of untreated wastewater (Krogseth et al., 2017a; Krogseth et al., 2017b), which could potentially be a source of DP and related compounds to Storvannet.

### 3.4 Chlorinated paraffins

#### 3.4.1 SCCPs and MCCPs

SCCPs and MCCPs are presented in Table 4, Table 5 and Supplementary Table SI.6, 7. Supplementary Figure SI.9 shows a PCA biplot which highlights the difference between MCCPs and SCCPs between compartments and lakes. Although only 57.1% of variation was explained by the first two principal components, the PCA biplot is still useful to identify clusters. Storvannet sediments and sticklebacks and benthic samples from both lakes are more influenced by MCCPs than SCCPs. While trout and char from both Storvannet and Takvannet are more influenced by SCCPs. Both SCCPs and MCCPs were detected in all sediment samples from Storvannet with median mass fractions of 63 ng/g dw (range: 46–115 ng/g dw) and 77 ng/g dw (range of 66–137 ng/g dw) for ΣSCCPs and ΣMCCPs, respectively. Higher mass fractions of MCCPs relative to SCCPs agree with current emission profiles for CPs, where MCCP usage and emissions have increased in recent years due to regulatory scrutiny and regulation of SCCPs. The MCCP:SCCP ratio was relatively consistent, and in most cases >1 (0.9, 1.2, 1.5, and 1.6 in the four samples respectively). Overall, total SCCP tended to be at least one order of magnitude higher in Storvannet than in Takvannet (median: 0.661 ng/g dw, range: 0.399–5.281 ng/g dw), but due to the small sample size, this difference was not statistically significant (Wilcoxon rank sum test, W = 12, *p* = 0.057). While MCCPs were detected in samples from Storvannet, they were not detected in samples from Takvannet. This may be due to the higher molecular mass and therefore low degree of LRAT of MCCPs (Wu et al., 2019). The presence of MCCPs in Storvannet may therefore be a result of local use of products containing CPs (Li et al., 2021).

**TABLE 4:** Mass fractions of SCCP homologue groups (denoted by the carbon chain length) in sediments (ng/g dw) and biota (ng/g lw) in Storvannet and Takvannet, as well as the ∑SCCPs. N = number of samples analyzed, number between parentheses represents the number of samples that were above LOD. Mass fractions < LOD were not included in the median and ranges.

		∑C10			∑C11			∑C12			∑C13		∑SCCP	
Lake	n	Median	Range	n	Median	Range	n	Median	Range	n	Median	Range	Median	Range
Storvannet														
Sediment	4 (4)	6.37	3.580–11.61	(4)	29.16	21.99–55.99	(4)	21.89	17.59–39.02	(4)	5.965	2.980–9.580	63.32	46.26–115.2
Mollusc	1 (1)	27.6	-	(1)	118	-	(1)	52.8	-	(1)	108	-	307	-
Chironomid	1 (0)	-	-	(0)	-	-	(0)	-	-	(0)	-	-	-	-
Stickleback	4 (4)	14.2	10.5–101	(4)	47.4	27.2–187	(4)	52.8	25.3–177	(4)	76.2	36.4–230	190	99.4–695
Arctic char	10 (3)	2.55	0.15–31.4	(4)	3.03	1.87–147	(4)	7.07	0.34–130	(4)	4.09	0.14–99.9	27.9	10.9–817
Brown trout	11 (2)	3.94	0.45–7.42	(3)	4.34	1.09–28.3	(3)	3.13	3.06–50.9	(3)	2.09	2.03–32.2	19.9	12.5–238
Takvannet														
Sediment	5 (3)	0.072	0.041–0.101	(3)	0.325	0.225–1.885	(3)	0.251	0.129–3.296	(3)	0.008	0.003–0.013	0.661	0.399–5.281
Amphipods	1 (1)	11.5	-	(1)	9.54	-	(1)	2.41	-	(1)	2.853	-	26.3	-
Valvatidae	1 (1)	3.87	-	(1)	10.3	-	(1)	4.91	-	(1)	10.54	-	29.6	-
Lymnae	1 (1)	16.5	-	(1)	49.9	-	(1)	26.6	-	(1)	32.05	-	125	-
Mollusc	1 (1)	5.40	-	(1)	1.79	-	(1)	0.56	-	(1)	2.125	-	9.86	-
Chironomid	1 (0)	-	-	(0)	-	-	(0)	-	-	(0)	-	-	-	-
Stickleback	3 (3)	3.64	0.19–6.32	(3)	5.79	0.83–6.61	(3)	1.36	0.24–11.7	(3)	0.83	0.05–46.6	11.6	1.31–71.2
Arctic char	9 (2)	2.24	1.04–3.45	(2)	26.1	9.80–42.4	(2)	5.16	1.09–9.23	(1)	0.24	-	67.2	23.8–110
Brown trout	10 (3)	0.23	0.03–0.63	(4)	3.00	1.13–7.00	(4)	1.43	0.23–9.68	(1)	5.67	-	5.99	1.36–20.2

**TABLE 5:** Mass fractions of MCCP homologue groups (denoted by the carbon chain length) in sediments (ng/g dw) and biota (ng/g lw) in Storvannet and Takvannet, as well as the ∑MCCPs. N = number of samples analyzed, number between parentheses represents the number of samples that were above LOD. Mass fractions < LOD were not included in the median and ranges.

		∑C14			∑C15			∑C16			∑C17		∑MCCP	
Lake	n	Median	Range	n	Median	Range	n	Median	Range	n	Median	Range	Median	Range
**Storvannet**														
Sediment	4 (4)	41.86	37.09–68.88	(4)	24.65	19.27–45.40	(4)	9.419	7.410–19.99	(4)	1.921	1.280–4.410	77.30	66.18–136.7
Mollusc	1 (1)	14047	-	(1)	9365	-	(1)	6302	-	(1)	2041	-	31757	-
Chironomid	1 (0)	-	-	(0)	-	-	(0)	-	-	(0)	-	-	-	-
Stickleback	4 (4)	459	107–1588	(4)	154	35.5–498	(4)	45.0	9.77–147	(4)	6.61	1.38–28.7	1330	307–4527
Arctic char	10 (2)	857	202–1510	(2)	118	17.8–218	(2)	3.26	0.42–6.10	(0)	-	-	977	220–1735
Brown trout	11 (4)	213	51.9–2228	(4)	42.5	3.63–1493	(3)	3.19	0.38–1004	(1)	111.8	-	258	55.6–4837
**Takvannet**														
Sediment	5 (0)	<LOD	-	(0)	<LOD	-	(0)	<LOD	-	(0)	<LOD	-	<LOD	-
Amphipods	1 (1)	225	-	(1)	116	-	(1)	52.8	-	(1)	14.8	-	409	-
Valvatidae	1 (1)	572	-	(1)	277	-	(1)	105	-	(1)	8.76	-	963	-
Lymnae	1 (1)	4081	-	(1)	2735	-	(1)	1888	-	(1)	634	-	9340	-
Mollusc	1 (1)	202	-	(1)	92	-	(1)	24.1	-	(1)	2.28	-	320	-
Chironomid	1 (1)			(1)			(1)			(1)				
Stickleback	3 (3)	196	29.2–3004	(3)	142	5.70–2291	(3)	62.5	0.12–1013	(2)	103	3.70–203	404	35.0–6512
Arctic char	9 (3)	182	85.9–660	(2)	25.9	12.2–499	(2)	8.46	3.90–254	(2)	11.7	-	1149	39.9–15255
Brown trout	10 (4)	380	18.1–4837	(3)	315	10.8–4412	(3)	361	11.0–4404	(1)	892	-	212	106–1425

In Storvannet, CPs were only detected in a mollusk sample with a ∑SCCP 2.41 ng/g ww (307 ng/g lw) and 124 ng/g ww (31757 ng/lw) of ∑MCCPs. In Takvannet SCCPs and MCCPs were measured in 4 benthic organisms, with Lymnae having the highest mass fractions for both CPs (∑SCCPs: 1.38 ng/g ww (125 ng/g lw), ∑MCCPs: 102 ng/g ww (9340 ng/g lw)) > Amphipods (SCCPs: 0.34 ng/g ww (26.3 ng/g lw), MCCPs: 5.27 ng/g ww (409 ng/g lw) > Valvatidae (SCCPs: 0.16 ng/g ww (29.6 ng7g lw), MCCPs: 5.31 ng/g ww (963 ng/g lw)) > Mollusc (SCCPs: 0.05 ng/g ww (9.86 ng/g lw), MCCPs: 1.65 ng/g ww (320 ng/g lw)) (Table 4; Table 5; Supplementary Table SI.6, 7).

∑SCCPs were observed in sticklebacks from both lakes, Storvannet (median: 10.2 ng/g ww, 191 ng/g lw) and Takvannet (median: 0.6 ng/g ww, 11.6 ng/g lw). Interestingly, in Storvannet, mass fractions detected in trout and char were lower than those found in Sticklebacks, while comparable mass fractions were found among all fish in Takvannet. Median mass fractions of ∑MCCPs in sticklebacks were 1331 ng/g lw (range: 308–4528 ng/g lw) in Storvannet and 404 ng/g lw (range: 35.0–6513 ng/g lw) in Takvannet, however they were not significantly different (Wilcoxon, W = 7, *p* = 0.86). For top predatory fish, the detection frequency for MCCPs was low for both trout (40%) and char (<30%) in both lakes. In Storvannet, ∑MCCPs mass fractions in trout with values above LOD ranged from 56 to 4838 ng/g lw (median: 258 ng/g lw) and in Takvannet they ranged from 106–1425 ng/g lw (median: 212 ng/g lw). These mass fractions were not significantly different between lakes (Wilcoxon, W = 7, *p* = 0.89). For char, ∑MCCPs in Storvannet ranged from 220 to 1735 ng/g lw (median: 978 ng/g lw), and in Takvannet from 39 to 15255 ng/g lw (median: 1149 ng/g lw). However, this maximum value is from one individual with an extremely low lipid content. Again, mass fractions were not significantly different between lakes (Wilcoxon, W = 5, *p* = 0.4). Within lakes there were no significant differences between char and trout (Storvannet: W = 3, *p* = 0.8; Takvannet: W = 8, *p* = 0.629). Although nonparametric tests were performed, results should be interpreted with caution due to the very small number of samples with values above LOD. Nevertheless, among the small number of fish with mass fractions above LOD, the medians were very similar in the two lakes. This was unexpected due to the proximity of Storvannet to an urban area. Similar to SCCPs, MCCPs did not show an increase in mass fractions with increasing δ ^15^N. The ∑SCCPs observed in trout in this study are only similar to those found in trout from Lake Erie (12 ± 7 ng/g lw) but are low in comparison to those found in trout from other Canadian lakes that ranged from 22 to 288 ng/g lw (Basconcillo et al., 2015). In comparison to char from Lake Ellasjøen on Bear Island (∑SCCPs 7–27 ng/g ww) (Katsoyiannis, 2013), both lakes from this study had also lower mass fractions. Comparison to other studies should be done with caution as different analytical and quantification methodologies may not be comparable (Pellizzato et al., 2009; van Mourik et al., 2020). Dick et al. (2010) observed similar results in Canada, where mass fractions in sticklebacks were higher than those in Arctic char. This is probably a reflection of sticklebacks feeding on benthic organisms.

BSAF values for SCCPs and MCCPs are shown in Supplementary Figures SI.10–12. BSAF values greater than 1 were found for several homologues in Storvannet benthos (C_10_Cl_6_, C_10_Cl_7_, C_11_Cl_6_, C_11_Cl_7_, C_12_Cl_5_, C_12_Cl_6_, C_12_Cl_7_, C_13_Cl_6_, C_13_Cl_7_, C_13_Cl_8_) and sticklebacks (C_10_Cl_6_, C_10_Cl_7_, C_11_Cl_6_, C_12_Cl_8_, C_13_Cl_7_, C_13_Cl_8_, C_13_Cl_9_), and for only a few homologues in Takvannet benthos (C_10_Cl_9_, C_11_Cl_9_, C_12_Cl_9_, C_13_Cl_10_), sticklebacks (C_12_Cl_9_, C_13_Cl_10_) and char (C_10_Cl_9_, C_11_Cl_9_, C_11_Cl_10_). BSAF for MCCPs could only be estimated for organisms in Storvannet, and the only benthic organism was not included due to its very low lipid content. BSAF values were above 1 also for several MCCP homologues in sticklebacks (C_14_Cl_8_, C_14_Cl_9_, C_14_Cl_10_, C_15_Cl_10_, C_16_Cl_10_), trout (C_14_Cl_5_, C_14_Cl_6_, C_15_Cl_5_, C_15_Cl_6_, C_15_Cl_7_, C_16_Cl_5_, C_16_Cl_6_, C_17_Cl_7_) and char (C_14_Cl_8_, C_14_Cl_9_, C_14_Cl_10_). Previous studies have shown a negative correlation between BSAFs and log *K*_ow_ (Sun et al., 2017; Huang et al., 2019), suggesting that bioaccumulation of SCCPs decreases with increasing log *K*_ow_ (Sun et al., 2017). While there was no evidence for such a relationship in our study (see Fig SI 10-12), this may be a result of the small sample size.

Although mass fractions of both SCCPs and MCCPs were low in this study, their presence in sub-Arctic lakes reflects that CPs can undergo LRAT (Wu et al., 2019; Jiang et al., 2021). Our results are in agreement with previous studies where higher mass fractions of CPs have been observed close to urban areas with industrial and anthropogenic activity (Li et al., 2021).

#### 3.4.2 CP homologue and congener group patterns

**FIGURE 4:**
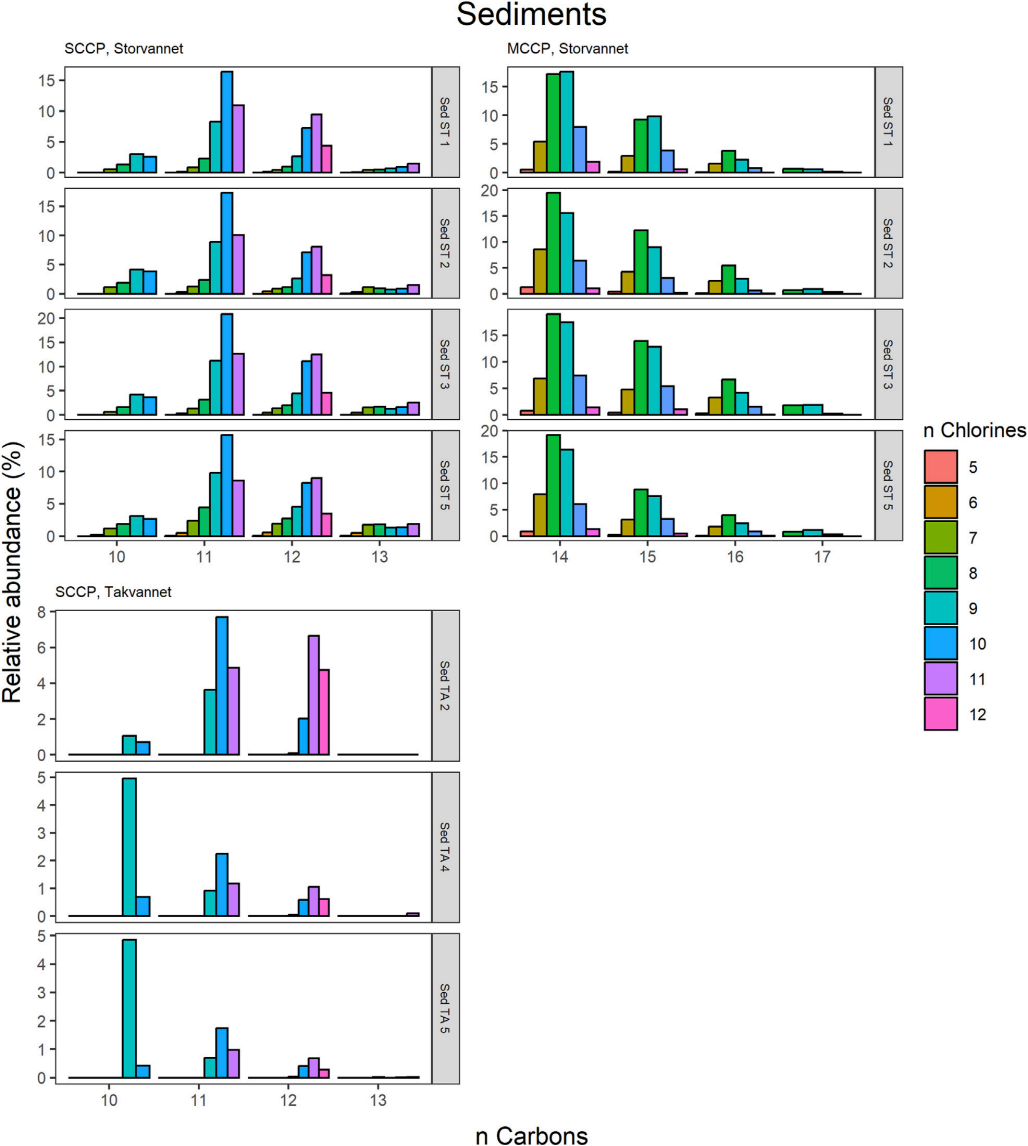
Relative abundance profiles of SCCP and MCCP homologues in sediment samples from Storvannet and Takvannet.

**FIGURE 5:**
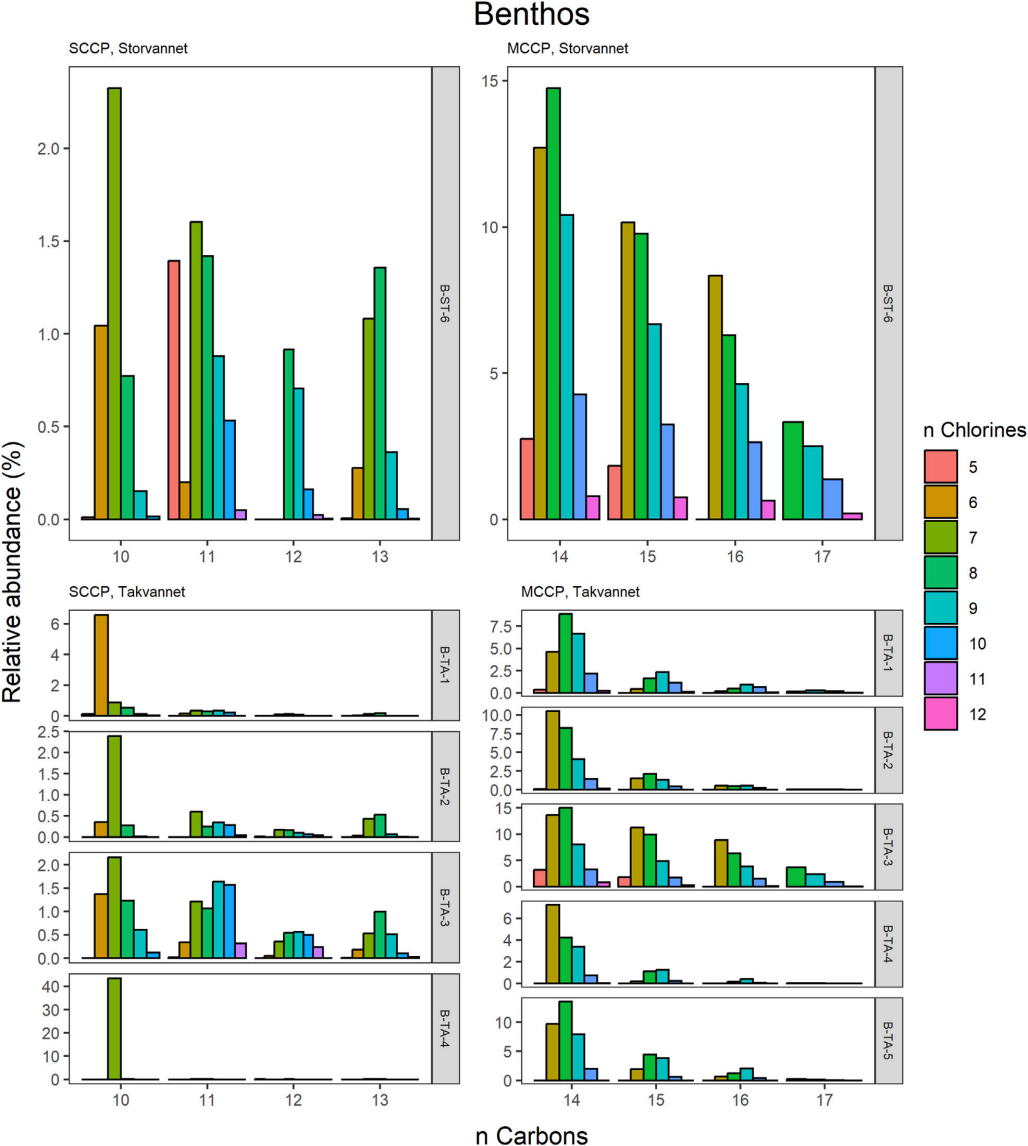
Relative abundance profiles of SCCP and MCCP homologues in benthic samples from Storvannet and Takvannet.

**FIGURE 6:**
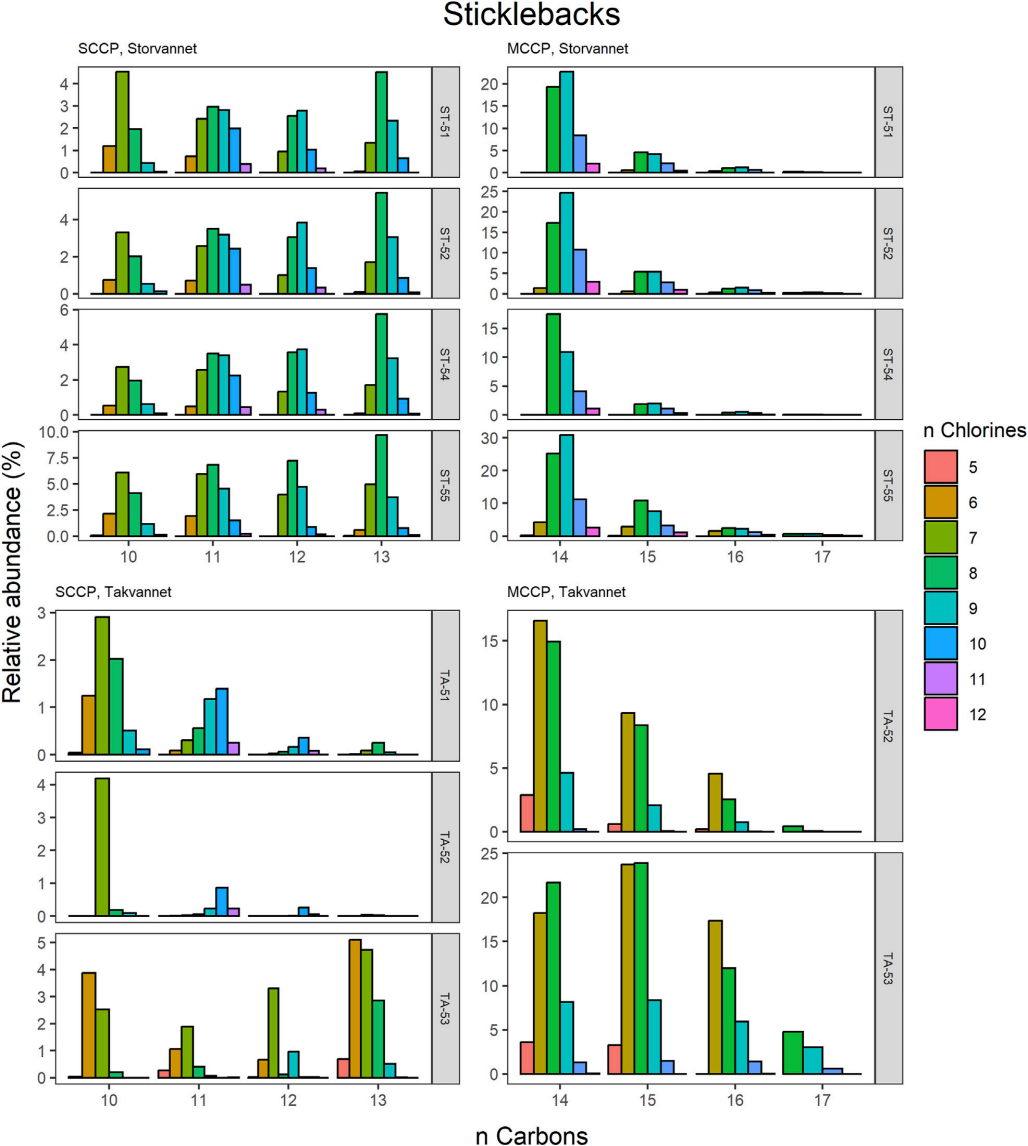
Relative abundance profiles of SCCP and MCCP homologues in three-spined stickleback samples from Storvannet and Takvannet.

**FIGURE 7:**
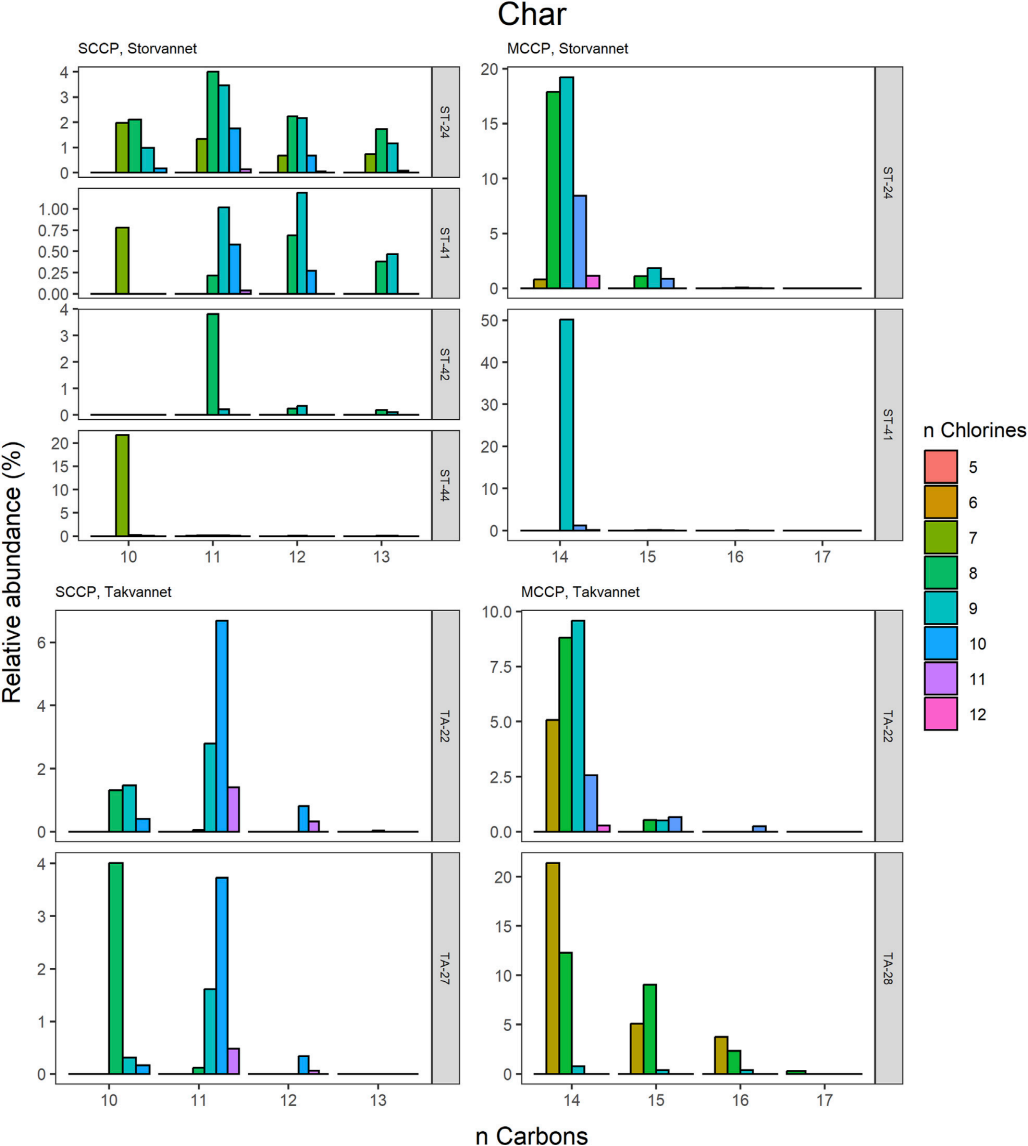
Relative abundance profiles of SCCP and MCCP homologues in Arctic char from Storvannet and Takvannet.

**FIGURE 8:**
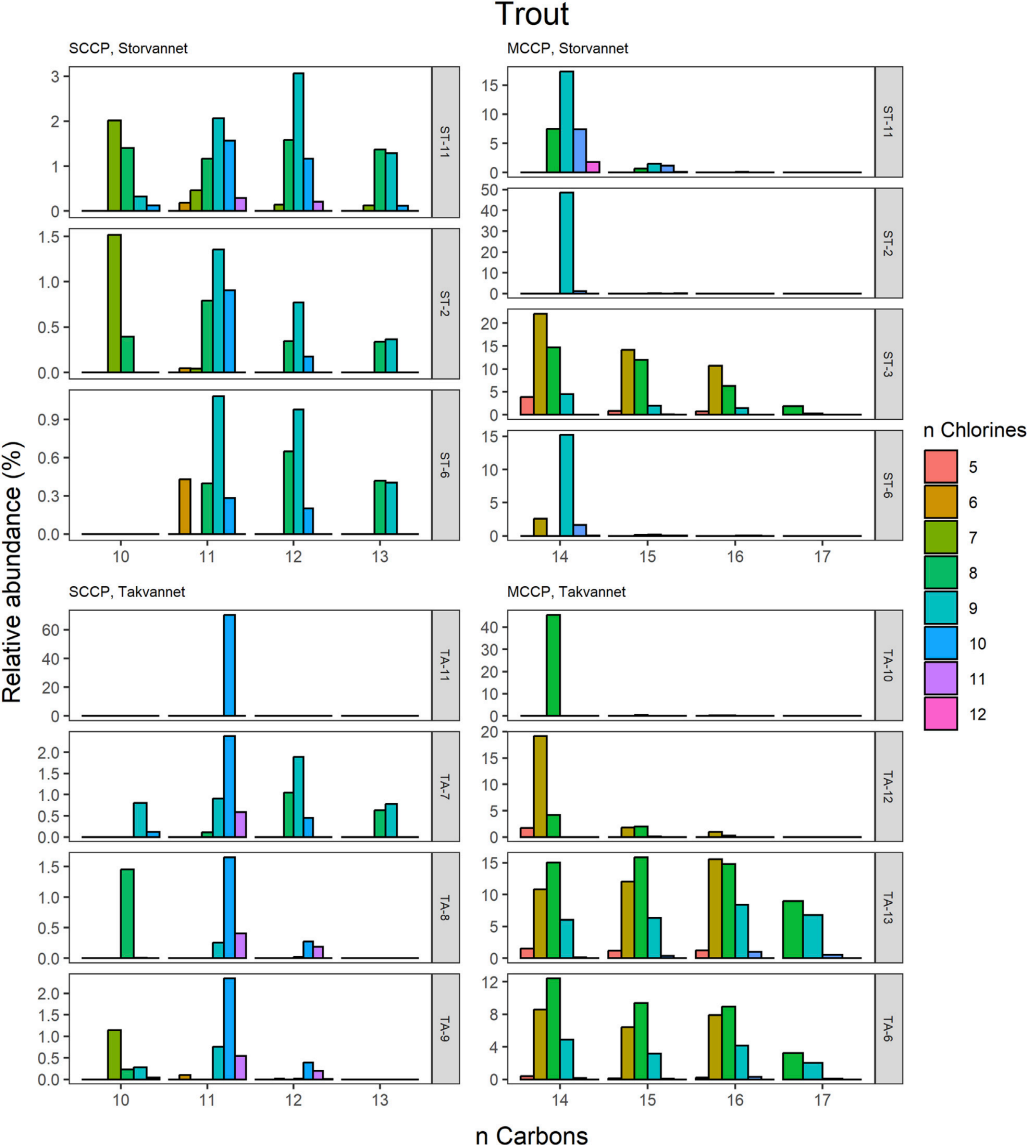
Relative abundance profiles of SCCP and MCCP homologues in Brown trout from Storvannet and Takvannet.

The congener group patterns for both SCCPs and MCCPs are provided in Figures 4–8. The patterns observed in both lakes showed SCCP profiles dominated by higher chlorinated congener groups while the MCCPs showed consistency in their profiles with C_14_ being the prevalent carbon chain length. Within the sediment samples, the C_11_ and C_12_ homologues contributed highest to the sum of SCCPs in both Storvannet (47% ± 1.5% and 35% ± 2.5%, respectively) and Takvannet (47% ± 10% and 44% ± 16%, respectively). The most abundant SCCP congener groups in sediment were C_11,_Cl_9-11_ and C_12,_Cl_10-12_ (Figure 4). This is in good agreement with sediment core samples from a rural lake in Switzerland showing a C_11-12_ contribution of 66%–87% (mean: 79%) (Iozza et al., 2008). The SCCP profiles we observed are very different compared to those observed in the technical mixtures where congeners with Cl_5-8_ dominate in the SCCP 51% and 55% Cl and Cl_7-10_ in the SCCP 63% Cl. Similar findings, i.e., high chlorination (Cl_8-11_) SCCP profiles, were observed in rubber granulates (Brandsma et al., 2019). This profile is most likely caused by use of different technical CP mixtures available worldwide for use in product manufacturing, in addition to recycling of materials to produce new products containing various technical mixes (Krätschmer and Schächtele, 2019). With adoption of recent regulations on the use of SCCPs under the Stockholm convention, SCCPs present in the environment will represent a very different profile to those observed prior to regulations. This is either as a result of degradation or use/recycling of different technical mixtures for production of products which warrants further investigation.

The MCCPs were only detected in sediments from Storvannet (Section 4.2.1) with C_14_ as the major contributor (54% ± 2.4%), followed by C_15_, C_16_ and C_17_ (32% ± 1.8%, 12% ± 1.0% and 2.6% ± 0.6%, respectively). The MCCP profiles are in good agreement with sediment samples from the North and Baltic Sea (Hüttig and Oehme, 2006) with contributions of 45%–50% and 31%–40% for the C_14_ and C_15_ homologues and from Lake Thun in Switzerland (C_14_; 41%–64%, mean: 48%). The MCCP profiles observed in sediment are almost identical to those in benthic organisms from both Storvannet and Takvannet (52% ± 9.9%, 30% ± 1.4%, 15% ± 6.3% and 3.3% ± 2.8% for C_14-17_, respectively) (Figure 5), highlighting the importance of organism-sediment coupling in driving food chain exposure to MCCPs. The SCCPs, however, showed differences in profiles between the sediment and benthic organisms. Comparable to the sediment samples, the C_11_ homologue was found to be the most dominant in almost all organisms (ranging from 18% to 40%) but was followed by the contribution of the C_13_ homologue (11%–36%) instead of the C_12_ homologue (9%–21%). The Chironomidae in both lakes showed SCCP profiles deviating from the other organisms with the C_13_ chain length as major contributor (Storvannet: 95% and Takvannet: 70%).

The high contributions of C_13_ were also observed in stickleback in Storvannet with profiles very similar between the individuals (Figure 6). The relative proportion increased with increasing carbon length, with C_13_ being the most dominant group of the SCCPs (18% ± 1.3%). One stickleback sample in Takvannet showed a profile similar to those in Storvannet, while the other two showed profiles comparable to the sediment samples with dominance of the C_11_ homologue followed by the C_12_. This could be attributed to the fact that shorter and less chlorinated CPs will have a greater volatility and be more easily transported via LRAT. The MCCP profiles of the sticklebacks in Storvannet showed consistency among all samples with the C_14-15_ as most abundant homologues (70% ± 0.7% and 23% ± 0.6%, respectively). The composition of the MCCPs in sediment and biota were found to be a combination of the MCCPs with 52% and 57% Cl, implying that their occurrence within the environment is a result from production and use of materials containing these mixtures. There was no significant difference found between the SCCP profiles detected in the top predatory fish in Takvannet (Figure 7; Figure 8). The C_11_ and C_12_ homologues were found to be most abundant in the trout and char samples (68% ± 23% and 24% ± 14%, respectively), different from the profiles observed in the biota samples from Storvannet where C_13_ was observed as greatest contributor. The C_10_ homologues dominated in both fish, but with a much higher contribution in char (71% ± 20%) than in trout (40% ± 16%). The opposite was observed with C_13_ homologue, which had higher contribution in trout (6.6% ± 5.7%) than in char (0.3% ± 0.5%). No clear explanation could be found for this occurrence and therefore more research is needed to understand the CP homologue behavior.

In conclusion, we have shown the presence of emerging and legacy pollutants in two lakes in the Norwegian sub-Arctic. Our results confirm higher mass fractions in a lake close to an urbanized area (Storvannet). This suggests that Storvannet may receive not only long-range transport input but in addition has local sources of pollution. This is not unexpected, since these chemicals have been widely used as flame retardants in industrial applications and household products such as in electrical and electronic equipment. The fact that these pollutants were also detected (albeit in low mass fractions) in the pristine Takvannet lake, suggests that this lake receives contaminants via atmospheric transport. Increase in mass fractions with increasing δ^15^N was only observed for PCBs and Dec 602 in Takvannet. The lack of trophic magnification in Storvannet resembles the previously observed and modelled concentrations of cyclic volatile methyl siloxanes (cVMS) in this lake. This could be explained by the characteristics of both the physical environment and food web structure. Storvannet has a rapid water turnover, causing a non-equilibrium situation between water and sediments, resulting in the highest mass fractions being present in benthic invertebrates. Mass fractions of CPs and DPs were observed in sediments, benthic organisms and sticklebacks but were detected in only few samples in trout and char. Higher mass fractions of MCCPs over SCCPs agrees with current emission profiles for CPs, where MCCP use, and emissions have increased in recent years due to regulatory scrutiny and regulation on SCCPs. These results contribute to our understanding of mass fractions of legacy and emerging contaminants in lake ecosystems and provide evidence of both long-range and locally sourced contamination in the subarctic.

## Data Availability

The raw data supporting the conclusion of this article will be made available by the authors, without undue reservation.
