# Supplementary material for: Dechloranes and chlorinated paraffins in sediments and biota of two subarctic lakes
Source: Front Toxicol. 2024 May 16;6:1298231. doi: 10.3389/ftox.2024.1298231 (PMC11137240; doi:10.3389/ftox.2024.1298231)
Supplement: Supplementary file 1 [file DataSheet1.PDF]

Supplementary Information

Dechloranes and chlorinated paraffins in sediments and biota of two subarctic lakes

List of Figures and Tables

Figures

SI.1 Isotope data in samples from Storvannet and Takvannet.

SI.2a and 2b. Mass fractions of ΣPCB 7 and PCB 153 in sediment and biota from Storvannet and Takvannet.

SI.3 – Relationship between $\delta^{15}N$ and the logarithmic PCB-153 mass fractions for all samples in Takvannet and Storvannet

SI.4 – Relationship between $\delta^{13}C$ and the logarithmic PCB-153 mass fractions for all samples in Takvannet and Storvannet.

SI.5 – PCB Principal Component Analysis.

SI. 6 – BSAFs vs log K_{ow} for 7 PCBs in benthos, sticklebacks, trout and char.

SI. 7 - Principal Component Analysis, comparison of Dechlorane plus and dechloranes in sediments, benthos, sticklebacks, trout and char in Takvannet and Storvannet.

SI. 8 – BSAF values for Dechlorane Plus and Dechlorane 602 in benthos, sticklebacks, trout and char in Takvannet (left) and Storvannet (right).

SI. 9 - Principal Component Analysis, comparison of Chlorinated Paraffins in sediments, benthos, sticklebacks, trout and char in Takvannet and Storvannet.

SI. 10 – BSAFs vs log K_{ow} for SCCPs in Takvannet.

SI. 11 – BSAFs vs log K_{ow} for for SCCPs in Storvannet.

SI. 12 – BSAFs vs log K_{ow} for MCCPs in Storvannet.

Tables

SI.1 - Morphometrics of char and trout in Storvannet and Takvannet.

SI.2 - Summary of stable isotopes, lipid content and total organic carbon.

SI.3 - PCB mass fractions in sediments (ng/g OC) and biota (ng/g ww) in Storvannet and Takvannet.

SI.4 - Mass fractions of DP isomers measured in sediment (ng/g dw) and biota (ng/g ww) in Storvannet and Takvannet

SI.5 - Mass fractions of Dec 602 and 603 measured in sediment (pg/g dw) and biota (pg/g ww) in Storvannet and Takvannet.

SI.6 - Mass fractions of ∑SCCP homologue groups measured in sediment (ng/g OC) and biota (ng/g ww) in Storvannet and Takvannet.

SI.7 - Mass fractions of ∑MCCP homologue groups measured in sediment (ng/g OC) and biota (ng/g ww) in Storvannet and Takvannet.

Materials and Methods

Quality Control Procedures

All glassware used during fieldwork and laboratory procedures was washed and rinsed with acetone and cyclohexane. Glassware was then covered in foil and heated to 450°C for 8 hrs and transfered immediately to the cleanroom. Sodium sulphate and silica was heat treated at 600°C for 8 hrs. Diatomaceous earth sorbent was rinsed with Hexane:DCM (1:1) in a separation funnel and put on the horizontal mixer (Shaker SM 30) for 1 hour at a speed of 175-180 rpm. This procedure was repeated 3 times. Solvents were drained and diatomaceous earth was left to dry for 1 hour in the funnel before spreading it in a foil tray in the cleanroom, were it was left overnight to dry.

Solvents were purchased from Merck (Darmstadt, Germany) *n*-hexane (Suprasolv, purity ≥ 98 %), acetone (Suprasolv, purity ≥ 99.8 %), DCM (purity ≥ 99.8%) and sulfuric acid (95-97%).

Internal and Recovery Standards

Internal and recovery standards used were as follows: for dechlorane analyses internal standard $^{13}C_{10}$ Dechlorane plus syn and $^{13}C_{10}$ Dechlorane 602 were used and recovery standard $^{13}C_{12}$ PCB 159 (Cambridge Isotope Laboratories, Massachusetts, USA)

For chlorinated paraffins we used internal standard $^{13}C_{10}$ Hexachlorodecane, $^{13}C_{12}$ Octachlorododecane and recovery standard TCN (1,2,3,4-tetrachloronaphthalene) from Dr. Ehrenstorfer GmbH’s laboratory (Augsburg, Germany)

Limits of Detection (LODs) were calculated as three times the standard deviation of the blanks. A high proportion of samples fell below the detection limit but since we performed a non-parametric analysis such as Wilcoxon-Mann-Whitney test, the result will not be much affected by how data below the limit are handled. Thus, methodology of
Substitute value of LOD/2 was used (Giskeødegård and Lydersen, 2022) when performing statistical analyses.

Total organic matter and total organic carbon

Total organic matter (TOM) was determined by weighing approx. 10 g of wet sediment in a pre-weighed porcelain crucible. The sediment was oven dried at 105°C to constant weight and then heated to 495°C for 16 hours. The method is based on NS 4764:1980.

Analyses of total organic carbon (TOC) was carried out following the method DIN19539:2016-12. In brief, wet sediment is dried at 40°C for at least 24 hours, and approx. 0.2 g dried sample is weighed accurately into a crucible, which is then placed into the SoliTOC Cube sample compartment. Each sample is heated stepwise to temperatures of 400 °C, 600 °C and 900 °C, under a flow of Oxygen and Nitrogen. Organic carbon is transformed into CO_2 (gas), detected by NDIR (Near Infra Red). Abundance of CO_2 emitted corresponds to the amount of organic carbon in the sample.

Stable isotopes

Biota and sediments from both lakes were analysed for stable isotopes of carbon and nitrogen, and isotopic ratios were calculated ($\delta^{13}C$ and $\delta^{15}N$). The analyses were carried out at the University of California, Davis (UC Davis StableIsotope Facility, USA). Samples were freeze-dried, homogenized, weighed, and packed in tin capsules. Samples of sediments and benthic organisms likely to have high calcium carbonate content were acidified to remove inorganic carbon. These were analyzed in parallel with unacidified samples (used for $\delta^{15}N$ values and N content). Samples were analyzed using a PDZ Europa ANCA-GSL elemental analyzer interfaced to a PDZ Europa 20-20 continuous flow isotope ratio mass spectrometer (IRMS), (Sercon Ltd., Cheshire, UK). Long-term standard deviation at UC Davis is +/-0.2‰ for ^{13}C and 0.3‰ for ^{15}N. Stable carbon and nitrogen isotope values are expressed using delta notation, relative to international standards (Vienna PeeDee Belemnite for C, and atmospheric N for nitrogen) (Peterson and Fry, 1987).

Result and Discussion

PCBs

The results in Takvannet were not unexpected due to the characteristics of PCBs which are well known to biomagnify with increasing trophic level. Some studies have shown that

biomagnification can decrease with trophic enrichment of the water body (Bentzen et al., 1996, Larsson et al., 1992) which enriches the growth of phytoplankton which will transport dissolved contaminants from the water column to sediments via algae sinking (Guildford et al., 2008) and makes the contaminants less available to fish. The difference between lakes may also be explained by the lake characteristics such as water turnover and habitat volume which can play an important role in the contaminant bioaccumulation in fish (Guildford et al., 2008). Bioaccumulation in fish occurs via bioconcentration (which is the process by which pollutants dissolved in water are absorbed by the branchias) but most importantly by its diet. Thus, the habitat preferences and the carbon sources (littoral vs pelagic) will directly affect the PCB concentrations leading to differences between individuals and species (Lopes et al., 2011). Guildford et al. (2008) described how trout which had access to littoral habitat had lower concentrations of pollutants in comparison to trout relying more on food webs based on pelagic phytoplankton. This was also observed in this study. As shown in Figure SI.4 the concentrations of PCB 153 decrease with δ13C values corresponding to more littoral carbon sources. There was a clear difference in δ13C between char and trout in Takvannet ($W=15$, $p=0.013$), with trout tending to be more littoral than char, which may explain the slightly higher concentrations observed in char. However, in Storvannet, there was no significant differences between char and trout ($W=47$, $p=0.604$) which may also help explain the lack of differences between species in their contaminant loads.

In a study by Sánchez-Hernández and Amundsen (2015), a clear difference in strategies was observed between char and trout (areas used: littoral vs pelagic and resources utilized) to avoid competition. They also noticed that sticklebacks and trout tend to overlap in terms of areas used. This may explain why even if sticklebacks occupy a lower trophic level than char and trout their concentrations were not statistically lower in either of the lakes. Differences in lake volumes can have an effect on the use of littoral vs pelagic habitats, Storvannet is a shallow and small lake and thus the use of habitat between char and trout may not be as clear as in a deeper and larger lake such as Takvannet.

Biota – sediment accumulation factors (BSAFs) are used in a number of environmental applications, and commonly in risk assessments (Burkhard et al., 2004, Melwani et al., 2009). BSAFs are suitable to describe bioaccumulation of sediment contaminants in the aquatic food webs. For benthic organisms, the use of BSAFs relies on 2 principal assumptions, including the assumption that benthic organisms are in a steady state with surrounding sediments which are the primary source of contamination. (Melwani et al., 2009) and the lack of metabolism.

In organisms that are closely linked to the sediments such as benthic invertebrates the BSAF values range between 1 and 2, depending on the affinity of the chemical for sediment organic carbon and for lipids (Burkhard et al., 2004) . However, for benthic fish species, it is assumed that bioaccumulation comes primarily from the contaminated prey which is associated with sediments (either benthic organisms or smaller fish), while the uptake from water is considered to be very small (Melwani et al., 2009). For fish, BSAFs are influenced by the biomagnification related to the trophic level of the species, their capacity for chemical metabolism, the diet of the fish, and its home range (Burkhard et al., 2004) all of which vary with their ontogenetic development. Therefore, we should not expect the BSAFs of fish to be within the range between 1 and 2. Figure SI. 6, shows BSAFs for PCBs in benthos, sticklebacks, trout and char from both lakes as a function of the *n*-octanol water partition coefficients (K_{ow}). In Storvannet, BSAFs ranged from 0.18 to 1.33 in benthos, from 0.56 to 1.95 in sticklebacks, from 1.39 to 2.00 for char, while trout had the highest values (from 0.40 to 5.55). The corresponding values for Takvannet were slightly higher with values from 1.80 to 10.2 for benthos, from 1.35 to 12.7 for sticklebacks, while both char (range: 20.7 – 53.7) and trout (8.20 – 27.3) had the highest values. The BSAF values show a clear increase with increasing K_{ow} values. In addition, it highlights how the bioaccumulation is less pronounced in Storvannet than in Takvannet.

Figures

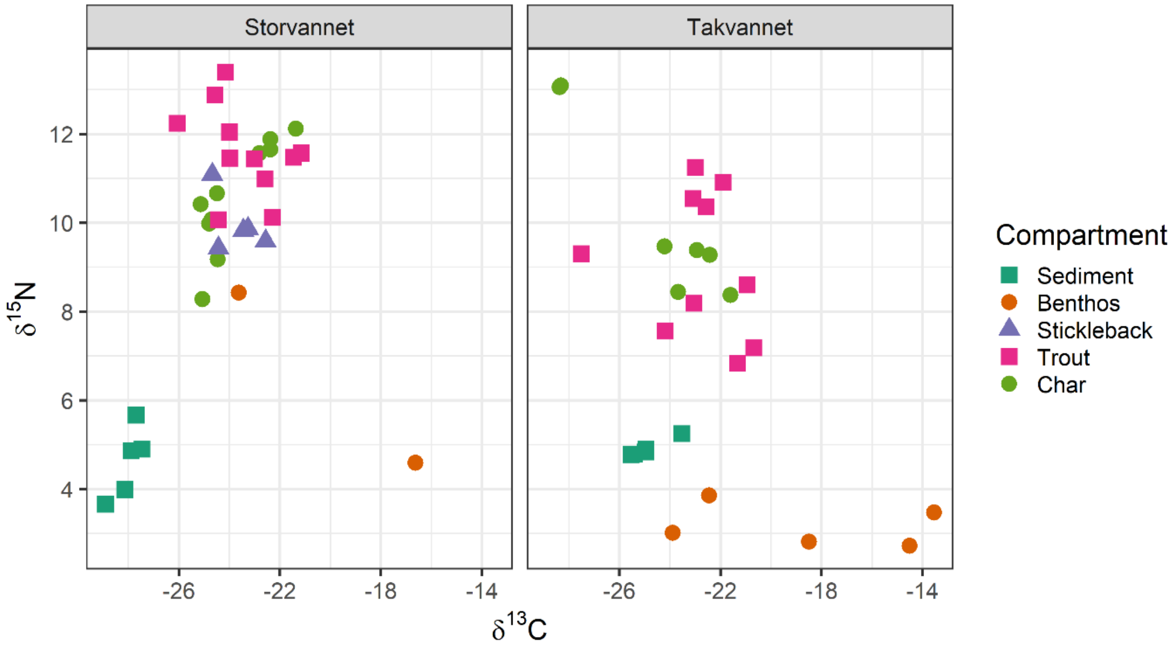

Figure SI.1 Isotope data in samples from Storvannet and Takvannet.

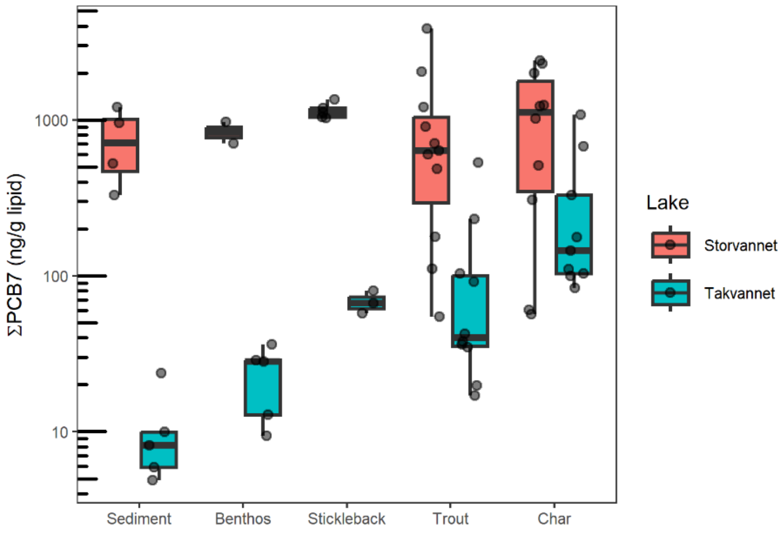

*Figure SI. 2a. ∑PCB 7 in sediment and biota from Storvannet and Takvannet. Dots represent each sample and black lines inside the boxes represent the median. The box covers the 25th to 75th percentile of the data. The upper whisker extends from the hinge to the largest value no further than 1.5 * IQR from the hinge (where IQR is the interquartile range, or distance between the first and third quartiles). The lower whisker extends from the hinge to the smallest value at most 1.5 * IQR of the hinge. Data beyond the end of the whiskers are "outlying" points and are plotted individually.*

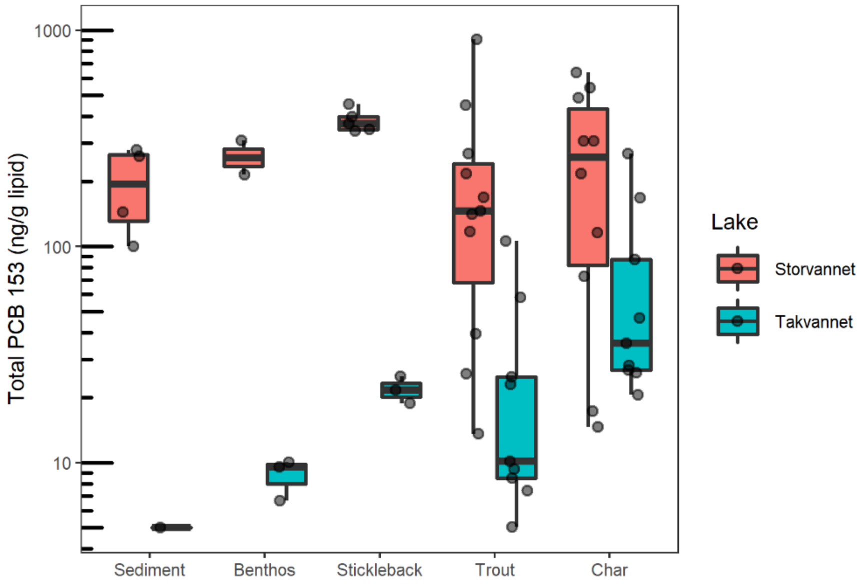

*Figure SI. 2b. Mass fractions of PCB 153 in sediment (ng/g OC) and biota (ng/g lw) from Storvannet and Takvannet. Dots represent each sample and black lines inside the boxes represent the median. The box covers the 25th to 75th percentile of the data. The upper whisker extends from the hinge to the largest value no further than 1.5 * IQR from the hinge (where IQR is the interquartile range, or distance between the first and third quartiles). The lower whisker extends from the hinge to the smallest value at most 1.5 * IQR of the hinge. Data beyond the end of the whiskers are "outlying" points and are plotted individually.*

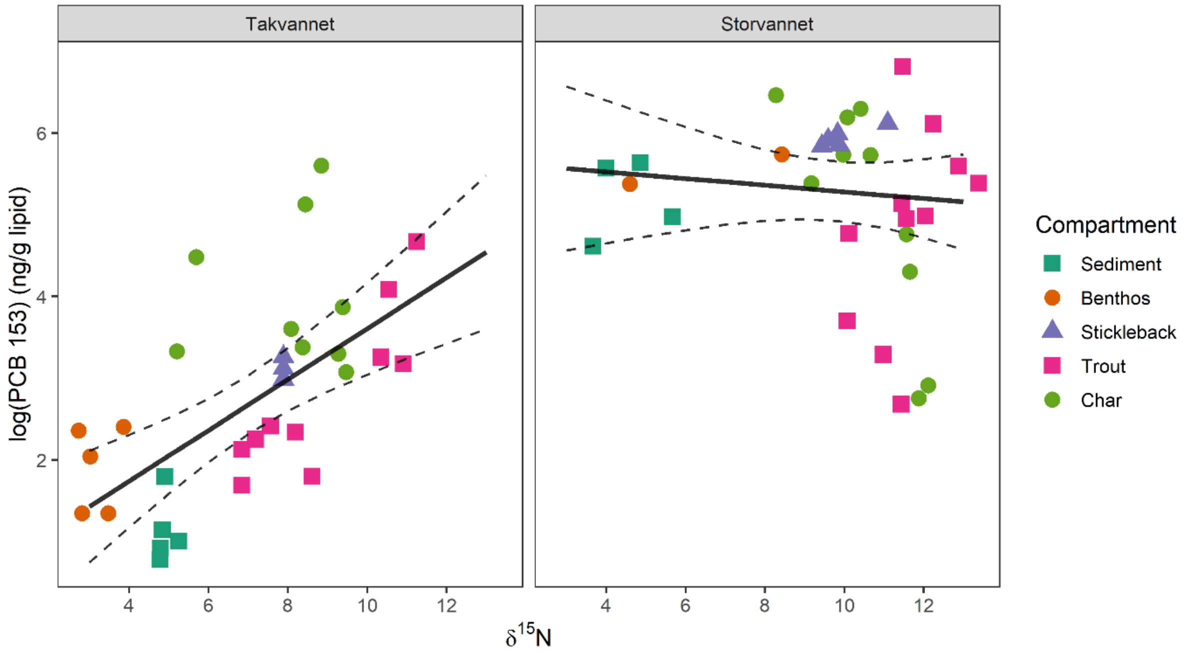

Figure SI. 3. Relationship between $\delta^{15}N$ and the logarithmic PCB-153 mass fractions for all samples in Takvannet and Storvannet. Solid black line represents the fit of the robust linear model, and the dash lines represent the 95% confidence interval of the fit.

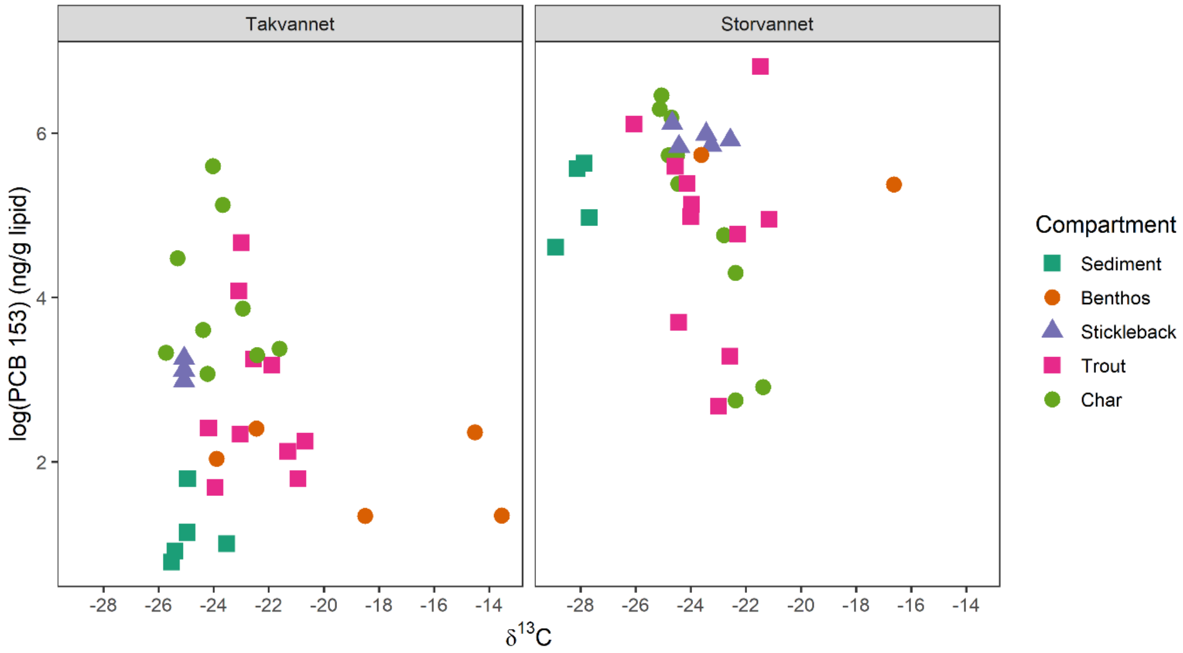

Figure SI. 4. Relationship between $\delta^{13}C$ and the logarithmic PCB-153 mass fractions for all samples in Takvannet and Storvannet.

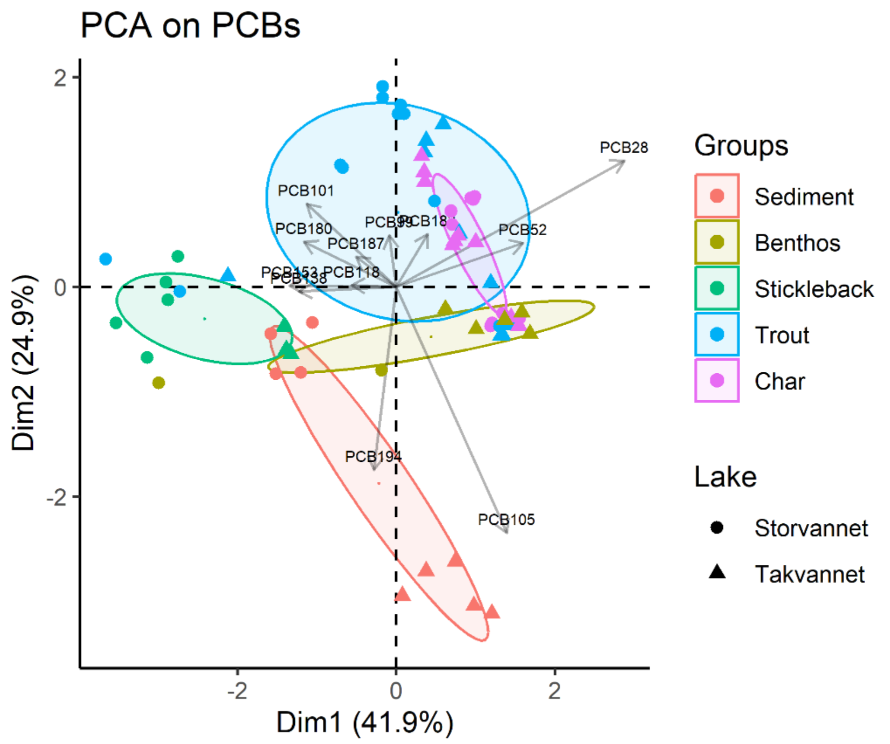

Figure SI. 5. Principal Component Analysis (PCA) for mass fractions of PCB congeners (ng/g OC or ng/g lw) in sediments, benthos, sticklebacks, trout and char in both Takvannet and Storvannet.

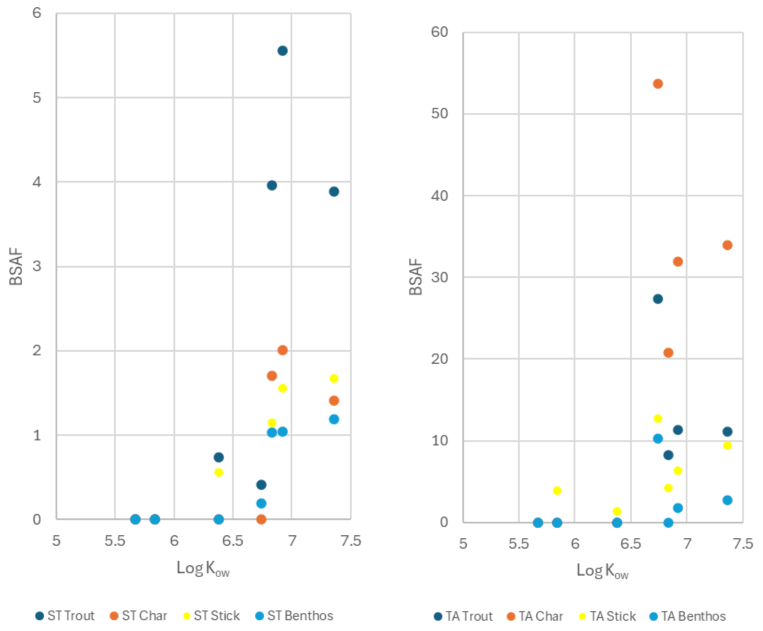

Figure SI. 6. BSAF values vs log K_{ow} for 7 PCB congeners in benthos, sticklebacks, trout and char for Storvannet (left) and Takvannet (right). Log K_{ow} values obtained from Hawker and Connell (1988)

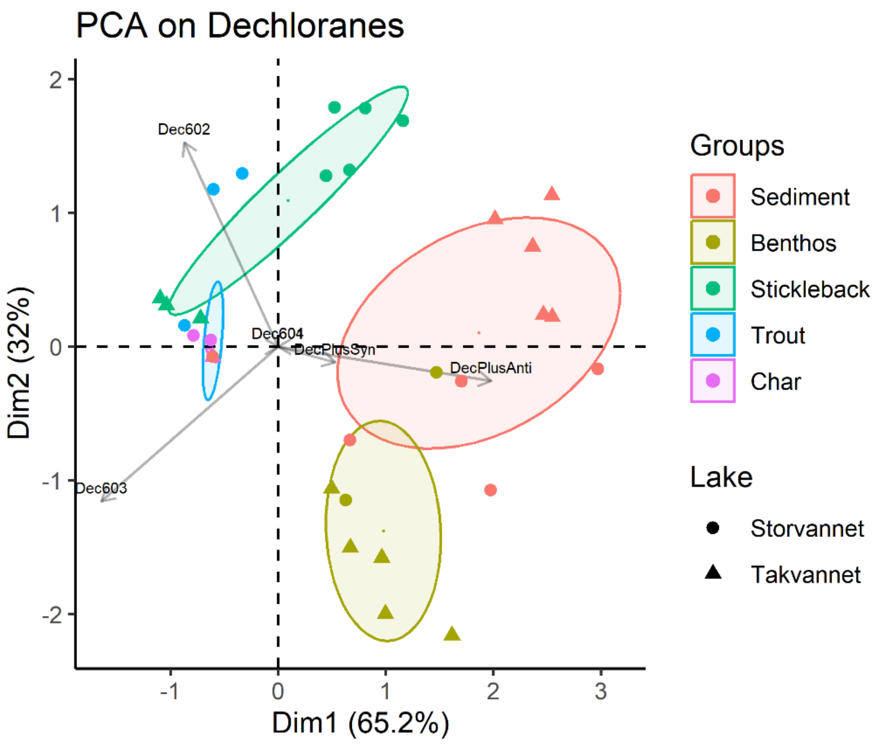

Figure SI. 7. Principal Component Analysis (PCA) for mass fractions of Dechloranes and Dechlorane Plus (ng/g lw or ng/g OC) in sediments, benthos, sticklebacks, trout and char in both Takvannet and Storvannet.

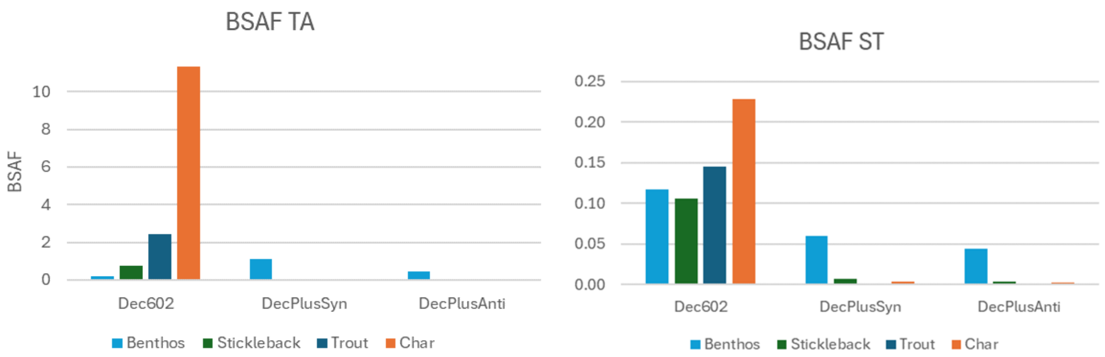

Figure SI. 8. BSAF values for Dechlorane Plus and Dechlorane 602 in benthos, sticklebacks, trout and char in Takvannet (left) and Storvannet (right).

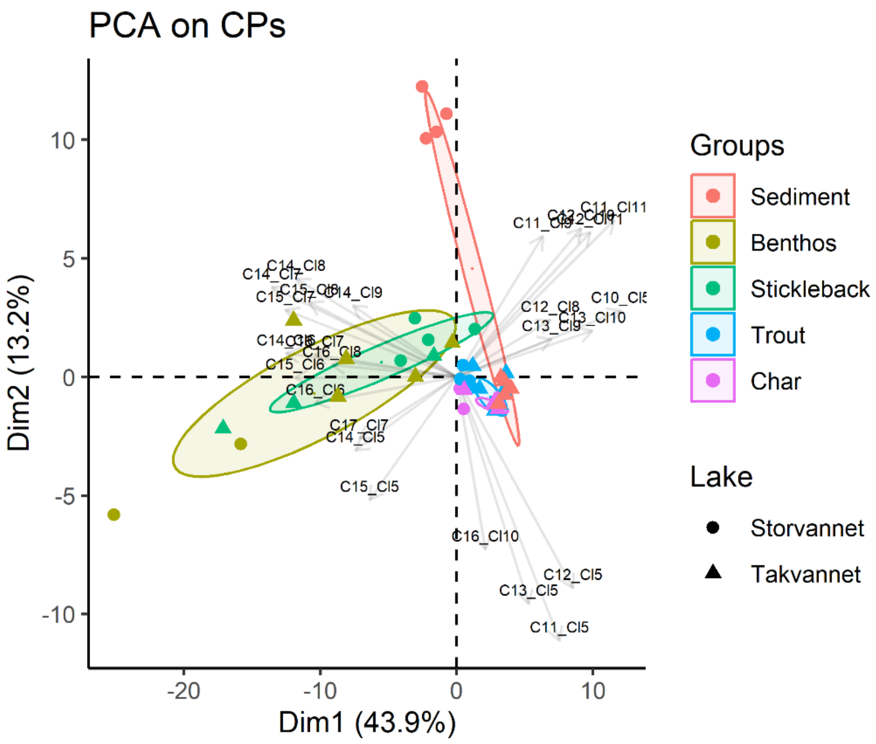

Figure SI. 9. Principal Component Analysis (PCA) for mass fractions of short-chain and medium-chain chlorinated paraffins (ng/g lw or ng/g OC) in sediments, benthos, sticklebacks, trout and char in both Takvannet and Storvannet.

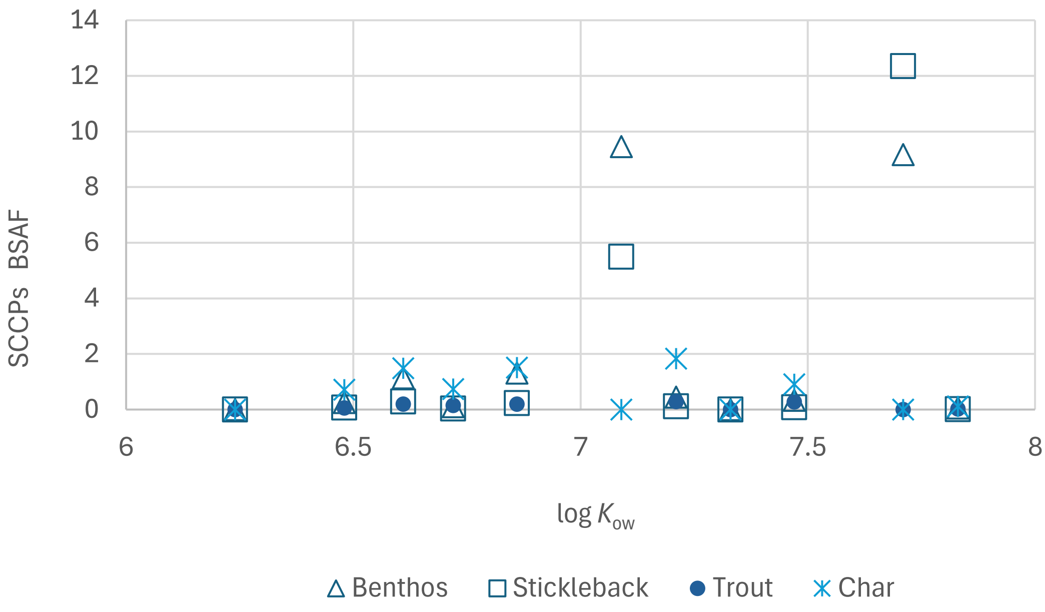

Figure SI. 10. BSAF values vs log K_{ow} of SCCP homologues in benthos, sticklebacks, trout and char from Takvannet. Log K_{ow} values obtained from Endo (2021).

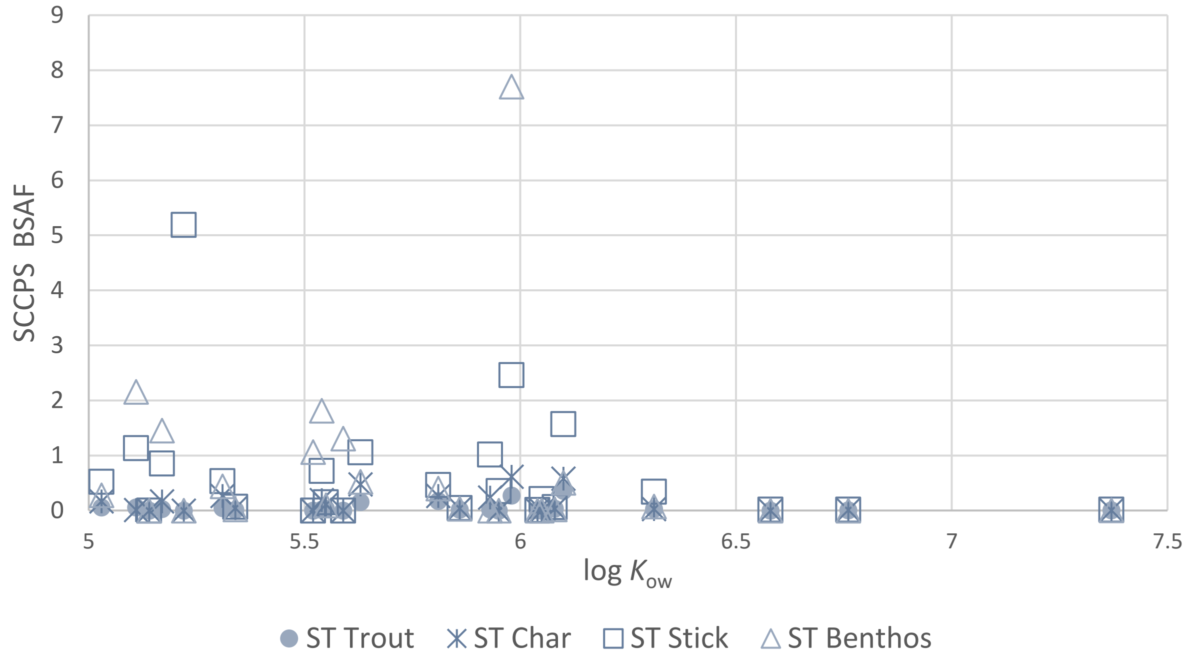

Figure SI. 11. BSAF values vs log K_{ow} of *SCCPs in benthos, sticklebacks, trout and char from Storvannet. . Log K_{ow} values obtained from Endo (2021).*

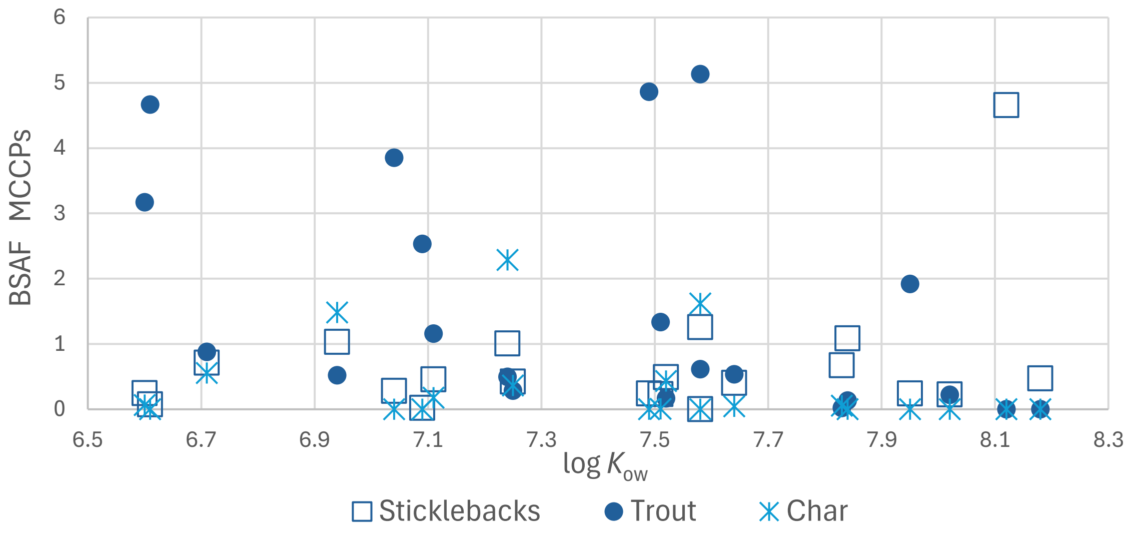

Figure SI. 12. BSAF values vs log K_{ow} of *MCCPs in sticklebacks, trout and char from Storvannet. . Log K_{ow} values obtained from Endo (2021).*

Tables

Table SI.1. Morphometrics of char and trout in Storvannet and Takvannet.

		Length (cm)		Weight (g)		Age	
Lake	N	Median	Range	Median	Range	Median	Range
Storvannet							
Trout	11	29.6	12.2 - 40.0	332	22.9 - 683	7	3 - 8
Char	10	27.5	14.2 - 44.0	286	29.3 - 763	7	2 - 9
Takvannet							
Trout	10	30	11.6 - 54.0	501	18.4 - 1650	5	3 - 9
Char	9	19.9	6.50 - 37.2	179	1.40 - 586	5	1 - 9

*Table SI.2. Summary of stable isotopes, lipid content and total organic carbon (TOC). * Values represent total organic carbon (TOC).*

		$\delta^{13}C$		$\delta^{15}N$		Lipid (%)/ TOC (mg/g)	
Lake	n	Median	Range	Median	Range	Median	Range
Storvannet							
Sediment	4	-28.15	-28.9 - -27.69	4.54	3.66 - 5.67	57.9*	43.5 - 68
Benthos	2	-20.12	-23.62 - -16.63	6.52	4.60 - 8.43	1.09	0.39 - 1.8
Stickleback	5	-23.68	-24.68 - -22.57	9.96	9.43 - 11.09	6.16	5.19 - 7.78
Trout	11	-23.43	-26.06 - -21.16	11.6	10.1 - 13.39	1.73	1.28 - 2.31
Char	10	-23.76	-25.13 - -21.37	10.6	8.28 - 12.12	1.11	0.51 - 3.41
Takvannet							
Sediment	5	-24.88	-25.53 - -23.53	4.91	4.78 - 5.25	27.4*	20.4 - 35.2
Benthos	5	-18.58	-23.89 - -13.55	3.18	2.73 - 3.86	1.16	0.51 - 2.33
Stickleback	3	-25.08	-25.08 - -25.08	7.89	7.89 - 7.89	6.92	5.54 - 8.54
Trout	10	-22.48	-24.19 - -20.69	8.91	6.84 - 11.25	1.82	0.51 - 3.26
Char	9	-23.96	-26.63 - -21.61	7.86	4.81 - 9.47	1.2	0.04 - 3.87

Classificatie: Intern

Table SI. 3. Mass fractions of ∑7-PCB and of PCB-153 in sediments (ng/g OC) and biota (ng/g ww) in Storvannet and Takvannet.

		∑ PCB 7		PCB 153	
Lake	n	Median	Range	Median	Range
Storvannet					
Sediment	4	741	328 - 1206	202	100 - 279
Benthos	2	10	3 - 17	3	0.8 - 6
Stickleback	5	68	62 -81	23	20 - 27
Trout	11	13	0.7 - 59	3	0.2 - 14
Char	10	9	0.6 - 21	2	0.2 - 6
Takvannet					
Sediment	5	9	5 - 28	2	1 - 5
Benthos	5	0.15	0.1 - 0.7	0.05	0.01 - 0.2
Stickleback	3	5	3 - 6	1.67	1 - 2
Trout	10	0.6	0.2 - 8	0.10	0.04 - 2
Char	9	1	0.3 - 6	0.30	0.1 - 2

Classificatie: Intern

Table SI. 4. Mass fractions of syn-DP, anti-DP, and DP total (syn-DP + anti-DP) measured in sediment (pg/g dw) and biota (pg/g ww) in Storvannet and Takvannet, as well as the calculated f_{anti} fraction. The numbers in parenthesis indicate the number of samples > LOD. Mass fractions < LOD are not included in medians and ranges. "na" indicates that the f_{anti} fraction and DP total mass fractions was not calculated because either of the DP isomers were detected < LOD.

Lake	n	syn-DP Median	syn-DP Range	n	anti-DP Median	anti-DP Range	n	f_{anti} Median	f_{anti} Range	n	DP Total Median	DP Total Range
Storvannet												
Sediment	4 (4)	564	191 - 2484	4 (4)	2669	1048 - 9997	4 (4)	0.82	0.78 - 0.85	4 (4)	3.23	1.24 - 12.5
Mollusc	1(1)	3	-	1(1)	10	-	1(1)	0.79	-	1(1)	13	-
Chironomid	1(1)	21	-	1(1)	59	-	1(1)	0.74	-	1(1)	80	-
Stickleback	5(5)	7	5 - 9	5(5)	13	-	5(5)	0.65	0.63 - 0.66	5(5)	20	-
Arctic char	10(1)	4	-	10(2)	5	3.9 - 5.2	10(1)	0.6	-	10(2)	6	3.9 - 8.7
Brown trout	10(0)	< LOD	-	10(2)	3	-	10(0)	na	-	10(2)	3	-
Takvannet												
Sediment	5(5)	7	2 - 13	5(5)	23	13 - 131	5(5)	0.80	0.77 - 0.91	5(5)	30	10 - 140
Amphipods	1(0)	< LOD	-	1(1)	1	-	1(0)	na	-	1(1)	1	-
Valvatidae	1(1)	1	-	1(1)	3	-	1(1)	0.76	-	1(1)	4	-
Lymnae	1(1)	4	-	1(1)	21	-	1(1)	0.86	-	1(1)	25	-
Mollusc	1(1)	4	-	1(1)	3	-	1(1)	0.43	-	1(1)	7	-
Chironomid	1(1)	4	-	1(1)	5	-	1(1)	0.54	-	1(1)	9	-
Stickleback	3(1)	3	-	3(3)	3	1 - 6	3(1)	0.64	-	3(3)	0.03	0.03 - 0.14
Arctic char	9(0)	< LOD	-	9(0)	< LOD	-	9(0)	na	-	9(0)	na	-
Brown trout	10(0)	< LOD	-	10(0)	< LOD	-	10(0)	na	-	10(0)	na	-

Table SI. 5. Mass fractions of Dec 602 and 603 measured in sediment (pg/g dw) and biota (pg/g ww) in Storvannet and Takvannet. The numbers in parentheses indicate the number of samples > LOD. Mass fractions < LOD are not included in medians and range.

	Dec 602			Dec 603		
Lake	n	Median	Range	n	Median	Range
Storvannet						
Sediment	4(4)	120	83 - 258	4(4)	161	56 - 222
Mollusc	1(1)	0.89	-	1(0)	<LOD	-
Chironomid	1(1)	6.26	-	1(1)	4.13	-
Stickleback	5(5)	14.6	8.13 - 25.3	5(5)	1.07	0.43 - 1.68
Arctic char	10(7)	4.9	1.0 - 5.6	10(0)	<LOD	-
Brown trout	11(8)	6.8	2.5 - 10.1	11(2)	1.6	1.4 - 1.7
Takvannet						
Sediment	5(5)	2.50	1.21 - 5.78	5(5)	0.28	0.17 - 1.62
Amphipods	1(1)	0.10	-	1(0)	<LOD	-
Valvatidae	1(1)	0.16	-	1(0)	<LOD	-
Lymnae	1(1)	0.28	-	1(0)	<LOD	-
Mollusc	1(1)	0.13	-	1(0)	<LOD	-
Chironomid	1(1)	0.38	-	1(0)	<LOD	-
Stickleback	3(3)	5.86	3.71 - 7.52	3(0)	<LOD	-
Arctic char	9(7)	3.96	0.61 - 28.0	9(0)	<LOD	-
Brown trout	10(10)	1.4	0.65 - 14.21	10(0)	<LOD	-

Table SI. 4. Mass fractions of SCCP homologue groups (denoted by the carbon chain length) in sediments (ng/g OC) and biota (ng/ g ww) in Storvannet and Takvannet, as well as the ∑SCCPs Mass fractions. N = number of samples analyzed, number between parentheses represents the number of samples that were above LOD. Mass fractions < LOD were not included in the median and ranges.

		∑C10			∑C11			∑C12			∑C13		∑SCCP	
Lake	n	Median	Range	n	Median	Range	n	Median	Range	n	Median	Range	Median	Range
**Storvannet**														
Sediment	4(4)	132	54.7 -170	(4)	609	336 - 810	(4)	448	271 - 599	(4)	110	45.6 - 173	1328	708 - 1695
Mollusc	1(1)	0.109	-	(1)	0.466	-	(1)	0.208	-	(1)	0.425	-	2.415	-
Chironomid	1(0)	-	-	(0)	-	-	(0)	-	-	(0)	-	-	-	-
Stickleback	4(4)	0.817	0.700 - 5.808	(4)	2.527	2.119 - 10.77	(4)	2.819	1.969 - 10.14	(4)	4.069	2.831 - 13.20	10.17	7.734 - 39.93
Arctic char	10(3)	0.087	0.001 - 0.273	(4)	0.083	0.027 - 1.278	(4)	0.072	0.011 - 1.136	(4)	0.042	0.005 - 0.869	0.186	0.142 - 3.55
Brown trout	11(2)	0.062	0.010 - 0.113	(3)	0.099	0.018 - 0.431	(3)	0.071	0.051 - 0.775	(3)	0.046	0.035 - 0.490	0.226	0.104 - 1.808
**Takvannet**														
Sediment	5(3)	2.04	1.60 - 3.76	(3)	9.23	8.76 - 70.3	(3)	7.13	5.02 - 123	(3)	0.139	0.13 - 0.37	18.8	15.5 - 197
Amphipods	1(1)	0.148	-	(1)	0.123	-	(1)	0.031	-	(1)	0.037	-	0.338	-
Valvatidae	1(1)	0.021	-	(1)	0.057	-	(1)	0.027	-	(1)	0.058	-	0.163	-
Lymnae	1(1)	0.182	-	(1)	0.550	-	(1)	0.293	-	(1)	0.353	-	1.378	-
Mollusc	1(1)	0.028	-	(1)	0.009	-	(1)	0.003	-	(1)	0.011	-	0.051	-
Chironomid	1(0)	-	-	(0)	-	-	(0)	-	-	(0)	-	-	-	-
Stickleback	3(3)	0.202	0.017 - 0.422	(3)	0.321	0.071 - 0.441	(3)	0.075	0.020 - 0.781	(3)	0.046	0.004 - 3.112	0.644	0.112 - 4.757
Arctic char	9(2)	0.051	0.040 - 0.062	(2)	0.570	0.379 - 0.760	(2)	0.104	0.042 - 0.165	(1)	0.004	-	0.726	0.461 - 0.991
Brown trout	10(3)	0.005	0.001 - 0.010	(4)	0.073	0.023 - 0.16	(4)	0.027	0.005 - 0.208	(1)	0.123	-	0.108	0.028 - 0.435

Table SI. 7. Mass fractions *of MCCP homologue groups (denoted by the carbon chain length) in sediments (ng/g OC) and biota (ng/g ww) in Storvannet and Takvannet, as well as the ∑MCCPs* mass fractions. *N = number of samples analyzed, number between parentheses represents the number of samples that were above LOD.* Mass fractions *< LOD were not included in the median and ranges*.

	∑C14			∑C15			∑C16			∑C17			∑MCCP	
Lake	n	Median	Range	n	Median	Range	n	Median	Range	n	Median	Range	Median	Range
**Storvannet**														
Sediment	4(4)	852	568 - 1012	(4)	470	338 - 667	(4)	190	113 - 265	(4)	39.7	19.5 - 64.9	1553	1039 - 2010
Mollusc	1(1)	55.23	-	(1)	36.82	-	(1)	24.78	-	(1)	8.026	-	124.8	-
Chironomid	1(0)	-	-	(0)	-	-	(0)	-	-	(0)	-	-	-	-
Stickleback	4(4)	27.61	5.89 - 91.30	(4)	9.30	1.96 - 28.65	(4)	2.701	0.540 - 8.480	(4)	0.381	0.080 - 1.650	40.00	8.460 - 130.1
Arctic char	10(2)	7.321	1.500 - 13.15	(2)	1.016	0.132 - 1.899	(2)	0.028	0.003 - 0.053	(0)	< LOD	-	8.364	1.630 - 15.10
Brown trout	11(4)	3.433	0.870 - 40.21	(4)	0.651	0.080 - 26.95	(3)	0.049	0.010 - 18.13	(1)	2.019	-	4.107	0.960 - 87.31
**Takvannet**														
Sediment	5(0)	< LOD	-	(0)	< LOD	-	(0)	< LOD	-	(0)	< LOD	-	-	-
Amphipods	1(1)	2.904	-	(1)	1.494	-	(1)	0.68	-	(1)	0.190	-	5.268	-
Valvatidae	1(1)	3.156	-	(1)	1.527	-	(1)	0.579	-	(1)	0.048	-	5.311	-
Lymnae	1(1)	44.97	-	(1)	30.14	-	(1)	20.81	-	(1)	6.993	-	102.9	-
Mollusc	1(1)	1.039	-	(1)	0.475	-	(1)	0.124	-	(1)	0.012	-	1.649	-
Chironomid	1(1)	7.997	-	(1)	4.552	-	(1)	2.049	-	(1)	0.193	-	14.19	-
Stickleback	3(3)	16.710	1.620 - 200.1	(3)	12.18	0.32 - 153.0	(3)	5.338	0.007 - 67.65	(2)	6.964	0.316 - 13.61	34.55	1.940 - 434.9
Arctic char	9(3)	7.040	0.300 - 64.20	(2)	6.059	0.180 - 58.55	(2)	7.234	0.180 -58.45	(2)	12.50	3.760 - 21.24	22.21	0.65 - 202.4
Brown trout	10(4)	3.267	0.950 - 8.98	(3)	0.465	0.140 - 6.790	(3)	0.094	0.070 - 3.46	(1)	0.160	-	3.802	1.18 - 19.39

Classificatie: Intern

References

Bentzen, E., Lean, D. R., Taylor, W. D. & Mackay, D. 1996. Role of food web structure on lipid bioaccumulation of organic contaminants by lake trout (Salvelinus namaycush). *Canadian Journal of Fisheries and Aquatic Sciences,* 53**,** 2397-2407.

Burkhard, L. P., Cook, P. M. & Lukasewycz, M. T. 2004. Biota--sediment accumulation factors for polychlorinated biphenyls, dibenzo-p-dioxins, and dibenzofurans in southern Lake Michigan lake trout (Salvelinus namaycush). *Environ Sci Technol,* 38**,** 5297-305.

Endo, S. 2021. Refinement and extension of COSMO-RS-trained fragment contribution models for predicting the partition properties of C10–20 chlorinated paraffin congeners. *Environmental Science: Processes & Impacts,* 23**,** 831-843.

Giskeødegård, G. F. & Lydersen, S. 2022. Measurements below the detection limit. . *Tidsskrift for Den norske legeforening.*

Guildford, S. J., Muir, D. C., Houde, M., Evans, M. S., Kidd, K. A., Whittle, D. M., Drouillard, K., Wang, X., Anderson, M. R., Bronte, C. R., Devault, D. S., Haffner, D., Payne, J. & Kling, H. J. 2008. PCB concentrations in lake trout (Salvelinus namaycush) are correlated to habitat use and lake characteristics. *Environ Sci Technol,* 42**,** 8239-44.

Hawker, D. W. & Connell, D. W. 1988. Octanol-water partition coefficients of polychlorinated biphenyl congeners. *Environmental Science & Technology,* 22**,** 382-387.

Larsson, P., Collvin, L., Okla, L. & Meyer, G. 1992. Lake productivity and water chemistry as governors of the uptake of persistent organic pollutants in fish. . *Environ Sci Technol***,** 346-352.

Lopes, C., Perga, M. E., Peretti, A., Roger, M. C., Persat, H. & Babut, M. 2011. Is PCBs concentration variability between and within freshwater fish species explained by their contamination pathways? *Chemosphere,* 85**,** 502-8.

Melwani, A. R., Greenfield, B. K. & Byron, E. R. 2009. Empirical estimation of biota exposure range for calculation of bioaccumulation parameters. *Integr Environ Assess Manag,* 5**,** 138-49.

Peterson, B. J. & Fry, B. 1987. Stable Isotopes in Ecosystem Studies. *Annual Review of Ecology and Systematics,* 18**,** 293-320.

Sánchez-Hernández, J. & Amundsen, P.-A. 2015. Trophic ecology of brown trout (Salmo trutta L.) in subarctic lakes. *Ecology of Freshwater Fish,* 24**,** 148-161.
